# Reirradiation Options for Previously Irradiated Prostate cancer (RO-PIP): Feasibility study investigating toxicity outcomes following reirradiation with stereotactic body radiotherapy (SBRT) versus high-dose-rate brachytherapy (HDR-BT)

**DOI:** 10.1136/bmjopen-2022-068580

**Published:** 2022-11-08

**Authors:** Jim Zhong, Sarah Brown, Maria Serra, Pam Shuttleworth, Peter Bownes, Christopher Thompson, Rachel Reed, Kimberley Reeves, Michael Dubec, Damien McHugh, Cynthia Eccles, Robert Chuter, Yat Man Tsang, N Jane Taylor, Catharine West, David Buckley, Andrew Scarsbrook, Ananya Choudhury, Peter Hoskin, Ann Henry

**Affiliations:** 1 Leeds Institute of Medical Research, University of Leeds, Leeds, UK; 2 Department of Radiology, St James's University Hospital, Leeds, UK; 3 Clinical Trials Research Unit, University of Leeds, Leeds, UK; 4 Department of Clinical Oncology, The Christie NHS Foundation Trust, Manchester, UK; 5 Department of Clinical Oncology, St James's University Hospital, Leeds, UK; 6 Division of Cancer Sciences, The University of Manchester, Manchester, UK; 7 Christie Medical Physics and Engineering, The Christie NHS Foundation Trust, Manchester, UK; 8 Department of Clinical Oncology, Mount Vernon Cancer Centre, Northwood, UK; 9 Paul Strickland Scanner Centre, Mount Vernon Hospital, Northwood, UK; 10 Leeds Institute of Cardiovascular and Metabolic Medicine, University of Leeds, Leeds, UK

**Keywords:** Prostate disease, RADIOTHERAPY, Magnetic resonance imaging

## Abstract

**Introduction:**

Radiotherapy is the most common curative treatment for non-metastatic prostate cancer; however, up to 13% of patients will develop local recurrence within 10 years. Patients can undergo further and potentially curative treatment including salvage surgery, brachytherapy (BT), external beam radiotherapy, high-intensity focused ultrasound and cryotherapy. Systematic review shows that high-dose-rate (HDR) BT and stereotactic body radiotherapy (SBRT) have the best outcomes in terms of biochemical control and lowest side effects. The reirradiation options for previously irradiated prostate cancer (RO-PIP) trial aims to determine the feasibility of recruitment to a trial randomising patients to salvage HDR-BT or SBRT and provide prospective data on patient recorded toxicity outcomes that will inform a future phase III trial.

**Methods and analysis:**

The primary endpoint of the RO-PIP feasibility study is to evaluate the patient recruitment potential over 2 years to a trial randomising to either SBRT or HDR-BT for patients who develop local recurrence of prostate cancer following previous radiation therapy. The aim is to recruit 60 patients across 3 sites over 2 years and randomise 1:1 to SBRT or HDR-BT. Secondary objectives include recording clinician and patient-reported outcome measures to evaluate treatment-related toxicity. In addition, the study aims to identify potential imaging, genomic and proteomic biomarkers that are predictive of toxicity and outcome based on hypoxia status, a prognostic marker of prostate cancer.

**Ethics and dissemination:**

This study has been approved by the Yorkshire and The Humber—Bradford Leeds Research Ethics Committee (Reference: 21/YH/0305, IRAS: 297060, January 2022). The results will be presented in national and international conferences, published in peer-reviewed journals and will be communicated to relevant stakeholders. A plain English report will be shared with the study participants, patients’ organisations and media.

**Trial registration number:**

ISRCTN 12238218 (Amy Ackroyd NIHR CPMS Team).

**Strengths and limitations of this study**

- The reirradiation options for previously irradiated prostate cancer study is the first to assess the feasibility to randomise men to different salvage radiation treatments for radio-recurrent prostate cancer.
- Randomised radiation treatment allocation will prevent selection bias.
- Using patient-reported outcome measures to collect treatment toxicity, data will allow for more patient-centred care.
- The addition of translational components into study will reveal information on the systemic and local effect of prostate reirradiation and identify candidate radioresponse biomarkers for inclusion in a phase III trial.
- Due to the feasibility design of the study, it is not powered to assess for differences in outcome between stereotactic body radiotherapy and high-dose-rate brachytherapy.

## Introduction

Prostate cancer is the most common cancer in men in the UK with approximately 48 500 new diagnoses every year and this has increased over the last 10 years.^1^ Worldwide, prostate cancer accounts for over 1.2 million new cases and causes over 350 000 deaths (3.8% of all deaths caused by cancer in men) annually.^2^ Radiation therapy (RT) is the most common curative treatment for non-metastatic prostate cancer.^3 4^ Despite advances in diagnostic imaging, RT delivery techniques and dose-escalation strategies, treatment failure remains common.^5–7^ At 10 years, the biochemical failure rate following treatment for localised prostate cancer with RT alone is 41%.^8^ Following dose-escalated RT, the most common site of cancer recurrence is in the prostate with 11-year cumulative incidence of local recurrence 7.2% and 13% in intermediate, and high-risk National Comprehensive Cancer Network prostate cancer groups, respectively.^7^

Multiple salvage options are available for locally recurrent disease including prostatectomy, reirradiation (with brachytherapy (BT) or external beam radiotherapy (EBRT)) and other focal therapies such as high-intensity focused ultrasound and cryotherapy. The evidence on the long-term effectiveness and quality-of-life impact for these treatments is limited; however, reirradiation techniques are the safest and most effective out of the currently available salvage treatment options.^9–13^ Most of the published literature describes retrospective case series with heterogeneous methodologies and radiation treatment techniques with no high-level evidence for the superiority of any of the reirradiation approaches or prospective comparative studies. Only a small proportion of patients with locally recurrent prostate cancer following primary radiotherapy (15%–20%) undergo local salvage therapy according to the Cancer of the Prostate Strategic Urologic Research Endeavor Registry.^14^

Prostate BT involves the placement of sealed radiation sources into the prostate and offers the ability to deliver highly conformal high-dose radiation with a steep dose gradient and rapid fall off in dose, which minimises radiation toxicity to surrounding organs at risk, specifically the rectum and bladder.^15^ Advances in image-guided-targeted BT may allow for more precise and focused treatments.^16–18^ BT can be delivered either via a permanent low-dose rate seed implant (LDR-BT), or via high-dose-rate BT (HDR-BT), which uses a high-activity radiation source (eg, iridium-192) that is temporarily introduced into applicators that are placed within the prostate, typically over 1–3 fractions. HDR-BT is less susceptible to issues related to prostate oedema and seed migration that might complicate dosimetry following LDR-BT. Current evidence suggests that HDR-BT affords lower toxicity, increased tolerability with similar oncological control compared with LDR-BT.^19^

Previously, salvage EBRT techniques have been associated with higher rates of severe late toxicities and also poor local control.^20^ Stereotactic body radiotherapy (SBRT), involving the delivery of a high dose of radiation to a highly conformal target volume with a steep dose gradient in a small number of fractions, may have benefits. Potential advantages include increased sparing of normal tissues than other types of EBRT and being less invasive compared with BT.^21^ BT is also highly specialised and only available in specialist centres.

A prospective trial is required to describe the toxicity profiles for these two most promising options, HDR-BT and SBRT, to allow clinicians and patients to make an informed decision on the most appropriate salvage treatment choice and help inform a larger study with an efficacy endpoint. Strategies to personalise salvage treatment through finding predictive genomic and imaging biomarkers are also required to optimise treatment outcomes.

The reirradiation options for previously irradiated prostate cancer (RO-PIP) trial is the first prospective randomised trial to determine the feasibility of recruitment to a trial comparing SBRT and HDR-BT for locally recurrent prostate cancer and inform power calculations for a definitive randomised control trial (RCT). In addition, this feasibility trial will also quantify the impact on patient-reported outcome measures (PROMs), quantify longitudinal functional MRI changes and assess proteomic, immune and genomic biomarkers.

## Method

### Study design

**Figure 1:**
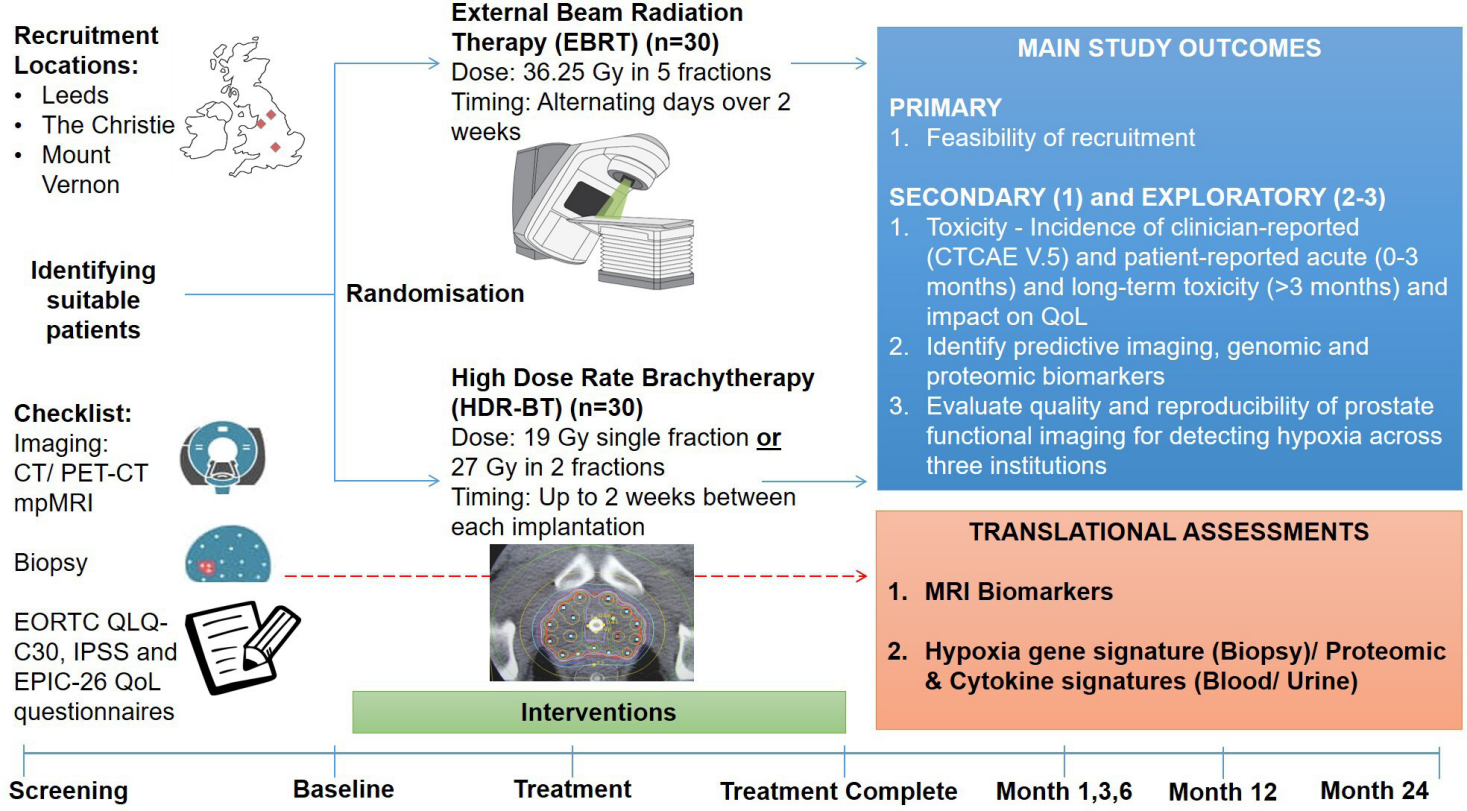
Schematic overview of study. CTCAE, Common Terminology Criteria for Adverse Events; EORTC QLQ, European Organization for the Research and Treatment of Cancer Quality of Life Questionnaire; EPIC, Expanded Prostate Cancer Index Composite; IPSS, International Prostate Symptom Score; mpMRI, multiparametric MRI; PET, positron emission tomography; QoL, quality of life.

The Standard Protocol Items: Recommendations for Interventional Trials (SPIRIT) checklist was adhered to when drafting the RO-PIP protocol.^22^ A completed SPIRIT checklist can be found with the trial protocol submission. A schematic overview of the study is shown in figure 1.

### Study setting

The planned study is a prospective two arm (HDR-BT and SBRT) randomised (1:1) feasibility trial aiming to recruit a total of 60 patients with locally recurrent prostate cancer across three tertiary referral oncology sites (Christie Hospital NHS Foundation Trust, Leeds Teaching Hospitals NHS Trust and Mount Vernon Cancer Centre, UK). The study will open for recruitment in September 2022, and the estimated primary recruitment completion date is in September 2024 and study completion date in September 2026.

### Patient and public involvement and engagement (PPIE)

We have sought input from the Leeds Cancer PPIE team and Leeds Radiotherapy user group into the protocol design, lay summary and patient information leaflet and have acted on the information provided.

### Consent and withdrawal

All participants will give written informed consent before entering the study and before any assessments or interventions related to the study are undertaken. Consent will be taken by the direct care clinical oncologist or a member of the RO-PIP research team, for example, clinical research fellow or research nurse. Optional consent will be sought for taking extra blood and urine samples for the translational study component. Participants are free to withdraw at any time, or at the discretion of the chief or principal investigator. In the event of withdrawal, any data collected up until that point will be kept and potentially included in any analyses.

### Eligibility criteria

The inclusion criteria are: (1) male individuals aged over 18 years; (2) histologically confirmed locally recurrent prostate cancer (following previous radiotherapy no less than 2 years ago); (3) no metastatic disease; (4) able and willing to provide an informed consent to participate; (5) WHO performance status 0–2; (6) reasonable urinary function (International Prostate Symptom Score (IPSS)<20 and Qmax>10 mL/s on flow tests); (7) greater than 10-year life expectancy.

The exclusion criteria are: (1) patients who are unfit for a general anaesthetic due to other comorbidities; (2) clinical or radiological evidence of metastatic prostate disease; (3) any patient with a medical or psychiatric condition that impairs their ability to give informed consent; (4) contraindication or intolerance of magnetic resonance scanning; (5) prior prostatectomy; (6) history of inflammatory bowel disease.

### Assignment of interventions

Following confirmation of written consent and eligibility, participants will be randomised into the trial by the Leeds Clinical Trials Research Unit (CTRU). Patients will be randomised on a 1:1 basis to receive either HDR-BT or SBRT. Patients will be randomised using stratified permuted blocks, stratified by recruiting site and previous androgen deprivation therapy (ADT) therapy. Randomisation will be performed centrally using the CTRU 9–5 telephone randomisation system. Authorisation codes and personal identification numbers, provided by the CTRU, will be required to access the randomisation system.

### Interventions

#### HDR brachytherapy

Two HDR-BT treatment schedules, either a single fraction 19 Gy treatment or 27 Gy in two fractions approximately 2 weeks apart, will be used to be decided by treating centre.

Gross tumour volume (GTV) will be delineated based on the intraprostatic lesion defined on the multiparametric MRI with or without additional diagnostic PET-CT information; the clinical target volume (CTV) is generated by applying an isotropic 3 mm margin constrained by the urethra (where applicable) and rectum. The CTV and planning target volume (PTV) are considered to be the same structures. The rectum, urethra and bladder should be contoured as organs at risk as per the European Society for Radiotherapy and Oncology (ESTRO) guidelines.^23^

#### Stereotactic body radiotherapy

Patients will receive five fractions of 7.25 Gy per fraction, which will be delivered alternate days over no more than 2 weeks to provide a total dose of 36.25 Gy. Radiotherapy may be delivered using CyberKnife, linear accelerator or MR-linear accelerator. Implanted prostate markers and SpaceOAR may be used as per centre standard of care.

GTV will be delineated based on the intraprostatic lesion defined on the multiparametric MRI with or without additional diagnostic PET-CT information; a CTV will be delineated comprising either the whole prostate or for focal treatment the GTV with a 3 mm margin constrained to the prostate boundaries. The CTV will then be grown by 3–5 mm (dependent on departmental policy and image guidance technique) to generate a PTV. The rectum, bladder, bowel loops (where appropriate) and femoral heads will be contoured as organs at risk.

#### Additional interventions

ADT may be initiated at the discretion of the treating oncologist but this must be started by the time of the first salvage radiotherapy treatment (at first fraction of SBRT or at HDR-BT).

#### Toxicity assessment

Clinician reported treatment toxicity will be summarised at each time point as the proportion of patients experiencing each toxicity, summarised by maximum grade experienced as per Common Terminology Criteria for Adverse Events (CTCAE) V.5.0.

#### PROMs assessment

Changes in patient-reported health-related quality of life (QoL)/PROMs will be assessed using the following validated questionnaires: Expanded Prostate Cancer Index Composite-26 (EPIC-26) (prostate cancer-related QoL and functional outcomes), European Organization for the Research and Treatment of Cancer Quality of Life Questionnaire (EORTC QLQ)-C30 (general QoL Score) and IPSS (urinary and sexual functional outcomes). The specific time points for these evaluations are:

- Baseline assessment (prior to salvage treatment).
- 1 month post-treatment completion.
- 3 months post-treatment completion.
- 6 months post-treatment completion.
- 12 months post-treatment completion.
- 24 months post-treatment completion.

The PROMs and quality-of-life assessments will not require a separate face-to-face meeting as these will be posted out to the participants. Follow-up after 2 years will be according to local policy.

#### Translational MRI assessment

All patients will have three multiparametric MRIs (including standard anatomical sequences and functional sequences), which will be paired with PROMs assessments at the same time points (at baseline and then post-treatment at 1 month and 1 year). The purpose of this imaging component is fourfold:

- To optimise a multiparametric MRI scanning protocol across three institutions incorporating intravoxel incoherent motion (IVIM), dynamic contrast-enhanced imaging and blood oxygenation level dependent sequences.
- To evaluate image quality and reproducibility of prostate functional imaging for detecting tissue perfusion and hypoxia.
- To investigate prostate and pelvic anatomical and functional imaging changes related to prostate reirradiation and how this relates to patient-reported toxicity side effects (determined by PROMs).
- To study the multiparametric MRI changes seen in the prostate in association with biopsy derived hypoxia-associated gene signature.

MRI scans done within the research study will be stored on the Leeds Teaching Hospitals Picture Archive and Communication System (PACS) server and on the local hospital PACS server where the images were obtained.

#### Translational biological assessment

The aim of this study component is to collect biological parameters that are prognostic and predictive markers of radiotherapy response and correlate this with imaging. From a biological stance the following sample collection will be relevant for assessing this:

- Tissue collection (prostate biopsy including original diagnostic and local recurrence sample) to measure the presence of a hypoxia-associated gene signature.
- Urine collection to measure the inflammatory response via damage-associated molecular patterns.
- Blood sample collection (20–30 mL) to measures changes in cytokine response following reirradiation and other proteomics analyses.

The long-term storage arrangement for the research data arising from these biological samples will follow The University of Manchester Biobank (site of processing for biological samples) good practice for research guidance on clinical samples. Participants will have the option to consent to making their biological samples available for future research. The biological sample research data will be stored for 20 years once the study has ended.

### Baseline and follow-up evaluation

Table 1 shows the full schedule of events.

**Table 1:** Reirradiation options for previously irradiated prostate cancer schedule of events

Item\Time point	Baseline	During RT	1-month FU	3-month FU	6-month FU	12-month FU	18-month FU	24-month FU
Informed consent	x							
Registration	x							
Testosterone	x							
Confirmation of eligibility	x							
Randomisation to BT or SBRT	x							
Pretreatment prostate biopsy sample requested and stored	x							
Collection of clinical history and outcome data	x							x
Radiotherapy (BT or SBRT) details	x	x						
PSA test	x			x	x	x	x	x
Functional prostate mpMRI	x		x			x		
PROM assessment (EPIC-26, EORTC QLQ-C30 and IPSS)	x		x	x	x	x		x
Research blood test/urine	x		x	x	x			
Clinician reported adverse events		x	x	x	x	x	x	x

BT, brachytherapy; EBRT, external beam radiotherapy; EPIC-26, Expanded Prostate Cancer Index Composite-26; FU, follow-up; IPSS, International Prostate Symptom Score; mpMRI, multiparametric MRI; PROM, patient-recorded outcome measure; PSA, prostate specific antigen; EORTC QLQ, European Organization for the Research and Treatment of Cancer Quality of Life Questionnaire; RT, radiotherapy.

### Outcomes

#### Primary outcome

1. Recruitment rates for the whole 24-month recruitment period will be reported overall and per recruiting site. The average recruitment rate per month and in total over the formal monitoring period will be reported.

The study recruitment period is 24 months. To show that patient recruitment targets for a phase III RCT can be met within an adequate timeframe, a ‘steady state’ of recruitment should be observed. In this feasibility study, formal monitoring of recruitment will begin from the start of the patient recruitment where an average of two patients per month must be randomised over the remaining recruitment period in order to demonstrate a ‘steady state’ of recruitment.

#### Secondary outcomes

1. Incidence of patient-reported acute (0–3 months) and long-term toxicity (>3 months) and impact on QoL determined by EPIC-26 (prostate cancer related QoL and functional outcomes), EORTC QLQ-C30 (general QoL score) and IPSS (urinary and sexual functional outcomes) measurements (key secondary endpoint).
2. Incidence of clinician-reported treatment toxicity as per CTCAE V.5.0.
3. Other feasibility endpoints include screening log summaries, treatment and questionnaire compliance, withdrawal rate and reasons for non-recruitment.

#### Exploratory outcomes

1. MRI biomarkers at 1 month and 1 year post-treatment predictive of toxicity based on PROMs.
2. Hypoxia levels based on a hypoxia associated gene signature obtained from the presalvage RT biopsy correlated with MRI biomarkers.
3. Changes in the levels of inflammatory cytokine signatures from urine and blood obtained at baseline and after reirradiation in relation to PROMs.
4. Multiple measures of image quality and reproducibility of prostate functional imaging (eg, diffusion coefficient values from IVIM sequences) for measuring tumour biology will be summarised.

### Sample size

This is an exploratory feasibility study, and there is no informative data in the published literature on which to base a sample size calculation. Therefore, a formal statistical power calculation has not been performed. Feasibility studies are not usually sufficiently powered to provide estimates of effect size, but instead aim to determine the feasibility of specific study aspects and to enable estimation of sample size parameters to inform future studies. For this feasibility study, we plan to recruit 60 patients in total (ie, 30 to each treatment arm) from three UK hospitals (Leeds Cancer Centre, The Christie and Mount Vernon Cancer Centre), all of which are high-volume tertiary prostate cancer treatment centres. This sample size has been informed by the National Institute of Health Research guidance on feasibility sample sizes and the toxicity outcome data from a recent systematic review.^13 24^ Few studies have evaluated PROMs feasibility, establishing the need for this trial.^25^ It is estimated that this number of participants will provide an adequate sample to estimate the toxicity rates for the key secondary endpoint in each arm (ie, 30 per arm).^26 27^ Recruiting 60 patients over 2 years, across 3 centres, would mean an average recruitment rate of 1 patient per centre per month. This information, combined with the estimated toxicity rates from the study, will be used to determine feasibility of a subsequent larger scale RCT.

### Recruitment

Overall, 60 patients will be recruited over a 24-month period, approximately 2–3 patients per month (across 3 sites).

### Reporting

No formal interim analysis will take place; however, a study report will be produced for review by the independent data monitoring and ethics committee (DMEC) approximately midway through the study. The aim of the report is to evaluate and monitor the key study objectives (ie, recruitment rates, number of participants taking up their treatment allocation), as well as expected adverse events (AEs) and serious AEs and the delivery time of HDR-BT or SBRT post randomisation.

### Data collection, management and analysis

#### Withdrawal of participants

Participants who withdraw their consent to the study will be taken off the study. The research team will keep any tissue, blood, urine samples and imaging data already collected and continue analysis (unless the patient requests the destruction of samples and data). Patients will be consented from the outset to continue collecting follow-up data even if patients are withdrawn from the trial, cannot tolerate MRI scans, are unable to continue treatment or do not complete all PROM time points.

#### Data management

Study data will be managed by the trial coordinator and research fellow under the supervision of the chief investigator and the study statistician.

Data stored on hospital computers will be password protected and in locked rooms in the local hospital radiotherapy and/or radiology (imaging data) departments, only accessible by the local research team. Each patient is assigned a unique patient study ID number at enrolment (based on site and trial number allocated during randomisation step), which will be used on all trial documentation. This pseudoanonymisation step will prevent the patient from being identified by those outside the local research team. The local investigator will keep a subject enrolment and identification log that contains the key to the code, that is, a record of the personal identification data linked to each patient study ID number.

In compliance with Good Clinical Practice guidelines and in accordance with the University of Leeds Code of Conduct and Research Ethics, the chief or local principal investigator will maintain all records regarding the conduct of the RO-PIP study. These will be securely archived for up to 20 years if required.

#### Statistical analysis

As this is a feasibility study, it will not involve hypothesis testing to identify whether the intervention has had an impact. Instead, data analysis will be descriptive and involve summary statistics. The analysis of all primary endpoint and all secondary endpoints relating to recruitment and withdrawals from the trial will take place at the end of the 24-month recruitment period. Final analysis of all other endpoint data will be carried out 6 months after the final participant has been randomised.

#### Future work

Demonstrating feasibility will facilitate a larger randomised study comparing salvage reirradiation options with ADT alone, the usual management option. This study would have primary endpoints of survival (overall and metastasis free). This is the first time that a hypoxia gene expression signature will be studied with hypoxia imaging in a prospective cohort of patients with radiorecurrent prostate cancer and could lead to the further studies investigating the introduction in clinical practice of tumour hypoxia testing (from biopsy and/or imaging) and the biological individualisation of radiotherapy. Given the large number of patients with prostate cancer who undergo radiotherapy each year, this would have a significant impact on personalised medicine in the UK.

#### Trial oversight

A trial management group will be convened for the study, consisting of the chief investigator, principal investigators (for each site), research fellow, trial administrator and research nurse. The group will meet monthly. The study sponsor (University of Leeds) will monitor the conduct of the trial. A trial report will be produced by the DMEC midway through the study

## Ethics and dissemination

This study has been approved by the Yorkshire and The Humber—Bradford Leeds Research Ethics Committee (Reference: 21/YH/0305, IRAS: 297060, January 2022).

The results will be presented in national and international conferences, published in peer-reviewed journals, publicised via social media channels such as twitter and will be communicated to relevant stakeholders. A plain English report will be shared with the study participants, patients’ organisations, PPIE groups and media.

## Supplementary Material

Reviewer comments

Author's manuscript
