# Supplementary material for: Reirradiation Options for Previously Irradiated Prostate cancer (RO-PIP): Feasibility study investigating toxicity outcomes following reirradiation with stereotactic body radiotherapy (SBRT) versus high-dose-rate brachytherapy (HDR-BT)
Source: BMJ Open. 2022 Nov 8;12(11):e068580. doi: 10.1136/bmjopen-2022-068580 (PMC9644357; doi:10.1136/bmjopen-2022-068580)
Supplement: Author's manuscript [file bmjopen-2022-068580.draft_revisions.pdf]

BMJ Open

BMJ Open is committed to open peer review. As part of this commitment we make the peer review history of every article we publish publicly available.

When an article is published we post the peer reviewers' comments and the authors' responses online. We also post the versions of the paper that were used during peer review. These are the versions that the peer review comments apply to.

The versions of the paper that follow are the versions that were submitted during the peer review process. They are not the versions of record or the final published versions. They should not be cited or distributed as the published version of this manuscript.

BMJ Open is an open access journal and the full, final, typeset and author-corrected version of record of the manuscript is available on our site with no access controls, subscription charges or pay-per-view fees (http://bmjopen.bmj.com).

If you have any questions on BMJ Open's open peer review process please email info.bmjopen@bmj.com

BMJ Open

Reirradiation Options for Previously Irradiated Prostate cancer (RO-PIP): Feasibility study investigating toxicity outcomes following reirradiation with stereotactic body radiotherapy (SBRT) vs. high dose-rate brachytherapy (HDR-BT)

Journal:	*BMJ Open*
Manuscript ID	bmjopen-2022-068580
Article Type:	Protocol
Date Submitted by the Author:	26-Sep-2022

Complete List of Authors:	Zhong, Jim; University of Leeds; St James's University Hospital, Diagnostic and Interventional Radiology<br>Brown, Sarah; University of Leeds, Clinical Trials Research Unit<br>Serra, Maria; The Christie NHS Foundation Trust, Department of Clinical Oncology<br>Shuttleworth, Pam; St James's University Hospital, Department of Clinical Oncology<br>Bownes, Peter; St James's University Hospital, Department of Clinical Oncology<br>Thompson, Christopher; St James's University Hospital, Department of Clinical Oncology<br>Reed, Rachel; The University of Manchester, Division of Cancer Sciences<br>Reeves, Kimberley; The University of Manchester, Division of Cancer Sciences<br>Dubec, Michael; The University of Manchester, Division of Cancer Sciences; The Christie NHS Foundation Trust, Department of Clinical Oncology<br>McHugh, Damien; The University of Manchester, Division of Cancer Sciences; The Christie NHS Foundation Trust, Department of Clinical Oncology<br>Eccles, Cynthia; The Christie NHS Foundation Trust, Department of Clinical Oncology; The University of Manchester<br>Chuter, Robert; The Christie NHS Foundation Trust, Department of Clinical Oncology; The University of Manchester, Division of Cancer Sciences<br>Tsang, Yat Man; Mount Vernon Cancer Centre, Department of Clinical Oncology<br>Taylor, N. Jane; Mount Vernon Hospital, Paul Strickland Scanner Centre<br>West, Catharine ; The University of Manchester Division of Cancer Sciences<br>Buckley, David; University of Leeds, Leeds Institute of Cardiovascular and Metabolic Medicine<br>Scarsbrook, Andrew; Leeds Teaching Hospitals NHS Trust, Department of Radiology; University of Leeds, Leeds Institute of Medical Research<br>Choudhury, Ananya; The Christie NHS Foundation Trust, Department of Clinical Oncology; The University of Manchester, Division of Cancer Sciences

Hoskin, Peter; Mount Vernon Cancer Centre, Department of Clinical Oncology; The University of Manchester, Division of Cancer Sciences
Henry, Ann; University of Leeds, Leeds Institute of Medical Research; St James's University Hospital, Department of Clinical Oncology

Keywords: Prostate disease < UROLOGY, RADIOTHERAPY, Magnetic resonance imaging < RADIOLOGY & IMAGING

SCHOLARONE™ Manuscripts

Reirradiation Options for Previously Irradiated Prostate cancer (RO-PIP): Feasibility study investigating toxicity outcomes following reirradiation with stereotactic body radiotherapy (SBRT) vs. high dose-rate brachytherapy (HDR-BT)

*Jim Zhong[1,2], Sarah Brown[3], Maria Serra[4], Pam Shuttleworth[2], Peter Bownes[2], Christopher Thompson[2], Rachel Reed[5], Kimberley Reeves[5], Michael Dubec[4,5], Damien McHugh[4,5], Cynthia Eccles[4,5], Robert Chuter[4,5], Yat Man Tsang[6], N. Jane Taylor[7], Catharine West[5], David L. Buckley[8], Andrew Scarsbrook[1,2], Ananya Choudhury[4,5], Peter Hoskin[4,5,6], Ann Henry[1,2]

1. Leeds Institute of Medical Research, University of Leeds, Leeds, UK
2. Leeds Cancer Centre, St James's University Hospital, Leeds Teaching Hospitals National Health Service (NHS) Trust, Beckett Street, Leeds, LS9 7TF
3. Leeds Institute of Clinical Trials Research (LICTR), University of Leeds, Leeds, UK
4. The Christie National Health Service (NHS) Foundation Trust, Manchester, UK
5. Division of Cancer Sciences, School of Medical Sciences, Faculty of Biology, Medicine and Health, The University of Manchester, The Christie Hospital, Manchester, UK
6. Mount Vernon Cancer Centre, Northwood, UK
7. Paul Strickland Scanner Centre, Mount Vernon Hospital, Northwood, Middlesex HA6 2RN
8. Leeds Institute of Cardiovascular and Metabolic Medicine, University of Leeds, Leeds, UK

*Corresponding author details

Dr Jim Zhong

Clinical Research Fellow and Radiology Registrar

Leeds Institute of Medical Research

St. James's University Hospital

University of Leeds

Beckett Street

Leeds

LS9 7TF

UK

Email: jim.zhong@nhs.net

For peer review only

Word count (excluding title, abstract and references) = 3267 (MAX 4000)

Acknowledgements: AC, PH, CE, CW are supported by the NIHR Manchester Biomedical Research Centre, UK. AS is supported by the Cancer Research UK (CRUK) Leeds Radiotherapy Research Centre of Excellence (RadNet C19942/A28832). JZ is supported by a CRUK Award (Leeds-Manchester Stella Erdheim Clinical PhD Fellowship – Grant Reference Number 95653117/95653118). This work was also supported by the Cancer Research UK Manchester Centre award [CTRQQR-2021\100010].

For peer review only

Abstract

Introduction

Radiotherapy is the most common curative treatment for non-metastatic prostate cancer, however up to 13% of patients will develop local recurrence within 10 years. Patients can undergo further and potentially curative treatment including salvage surgery, brachytherapy (BT), external beam radiotherapy (EBRT), high intensity focused ultrasound and cryotherapy. Systematic review shows that high dose rate (HDR) BT and stereotactic body radiotherapy (SBRT) have the best outcomes in terms of biochemical control and lowest side effects. The RO-PIP trial aims to determine the feasibility of recruitment to a trial randomising patients to salvage HDR-BT or SBRT and provide prospective data on patient recorded toxicity outcomes that will inform a future phase III trial.

Methods and analysis

The primary endpoint of the RO-PIP feasibility study is to evaluate the patient recruitment potential over 2 years to a trial randomising to either SBRT or HDR-BT for patients who develop local recurrence of prostate cancer following previous radiation therapy. The aim is to recruit 60 patients across 3 sites over 2 years and randomise 1:1 to SBRT or HDR-BT. Secondary objectives include recording clinician and patient reported outcome measures (PROMs) to evaluate treatment-related toxicity. In addition, the study aims to identify potential imaging, genomic and proteomic biomarkers that are predictive of toxicity and outcome based on hypoxia status, a prognostic marker of prostate cancer.

Ethics and dissemination

This study has been approved by the Yorkshire & The Humber - Bradford Leeds Research Ethics Committee (Reference: 21/YH/0305, IRAS: 297060, January 2022). The results will be presented in national and international conferences, published in peer-reviewed journals and will be communicated to relevant stakeholders. A plain English report will be shared with the study participants, patients' organisations and media. Trial registration number: ISRCTN 12238218

For peer review only

Article Summary

Strengths and limitations

- The RO-PIP study will be the first trial to assess the feasibility to randomise prostate cancer patients to different salvage radiation treatments for radio-recurrent prostate cancer.
- The trial will inform a future phase III randomised trial comparing survival and toxicity outcomes.
- Planned longitudinal functional magnetic resonance imaging (MRI) assessments will reveal information on the effect of radiotherapy on tumour biology and identify candidate radioresponse biomarkers for inclusion in a phase III trial.
- Longitudinal proteomic and immune marker analyses may reveal mechanistic insights into the systemic biological effect of reirradiation.
- Due to the feasibility design of the study, it is not powered to assess for differences in outcome between SBRT and HDR-BT.

Keywords

Prostate cancer; radiotherapy

For peer review only

Introduction

Prostate cancer is the most common cancer in males in the United Kingdom with approximately 48,500 new diagnoses every year and this has increased over the last 10 years [1]. Worldwide, prostate cancer accounts for over 1.2 million new cases and causes over 350,000 deaths (3.8% of all deaths caused by cancer in men) annually [2]. Radiation therapy (RT) is the most common curative treatment for non-metastatic prostate cancer [3,4]. Despite advances in diagnostic imaging, RT delivery techniques and dose-escalation strategies, treatment failure remains common [5–7]. At 10-years, the biochemical failure rate following treatment for localised prostate cancer with RT alone is 41% [8]. Following dose-escalated RT, the most common site of cancer recurrence is in the prostate with 11-year cumulative incidence of local recurrence 7.2%, and 13% in intermediate, and high risk National Comprehensive Cancer Network (NCCN) prostate cancer groups respectively [7].

Multiple salvage options are available for locally recurrent disease including prostatectomy, reirradiation (with brachytherapy (BT) or external beam radiotherapy (EBRT)) and other focal therapies such as high-intensity focused ultrasound (HIFU) and cryotherapy (CRYO). The evidence on the long-term effectiveness and quality of life impact for these treatments are limited, however reirradiation techniques are the safest and most effective out of the currently available salvage treatment options [10–14]. Most of the published literature describes retrospective case-series with heterogeneous methodologies and radiation treatment techniques with no high level evidence for the superiority of any of the re-irradiation approaches or prospective comparative studies. Only a small proportion of patients with locally recurrent prostate cancer following primary radiotherapy (15-20%) undergo local salvage therapy according to the Cancer of the Prostate Strategic Urologic Research Endeavor (CaPSURE) registry [9].

Prostate BT involves the placement of sealed radiation sources into the prostate and offers the ability to deliver highly conformal high dose radiation with a steep dose gradient and rapid fall off in dose which minimises radiation toxicity to surrounding organs at risk, specifically the rectum and bladder [15]. Advances in image-guided targeted BT may allow for more precise and focused treatments [16–18]. BT can be delivered either via a permanent low-dose rate seed implant (LDR-BT), or via high-dose rate BT (HDR-BT) which uses a high-activity radiation source (e.g. iridium-192) that is temporarily introduced into applicators that are placed within the prostate, typically over 1-3 fractions. HDR-BT is less susceptible to issues related to prostate oedema and seed migration that might complicate dosimetry following LDR-BT. Current evidence suggests that HDR-BT affords lower toxicity, increased tolerability with similar oncological control compared to LDR-BT [19].

For peer review only

Previously, salvage EBRT techniques have been associated with higher rates of severe late toxicities and also poor local control [20]. Stereotactic body radiation therapy (SBRT), involving the delivery of a high dose of radiation to a highly conformal target volume with a steep dose gradient in a small number of fractions may have benefits. Potential advantages include increased sparing of normal tissues than other types of EBRT, and being less invasive compared to BT [21]. BT is also highly specialised and only available in specialist centres.

A prospective trial is required to describe the toxicity profiles for these two most promising options, HDR-BT and SBRT, to allow clinicians and patients to make an informed decision on the most appropriate salvage treatment choice and help inform a larger study with an efficacy endpoint. Strategies to personalise salvage treatment through finding predictive genomic and imaging biomarkers are also required to optimise treatment outcomes.
The RO-PIP trial is the first prospective randomised trial to determine the feasibility of recruitment to a trial comparing SBRT and HDR-BT for locally recurrent prostate cancer and inform power calculations for a definitive RCT. In addition, this feasibility trial will also quantify the impact on patient reported outcome measures (PROMs), quantify longitudinal functional MRI changes and assess proteomic, immune and genomic biomarkers.

Method

Study Design

The Standard Protocol Items for Randomized Trials (SPIRIT) checklist was adhered to when drafting the RO-PIP protocol[22]. A completed SPIRIT checklist can be found with the trial protocol submission. A schematic overview of the study is shown in Figure 1.

Study Setting

The planned study is a prospective two arm (HDR-BT and SBRT) randomised (1:1) feasibility trial aiming to recruit a total of 60 patients with locally recurrent prostate cancer across three tertiary referral oncology sites (Christie Hospital NHS Foundation Trust, Leeds Teaching Hospitals NHS Trust and Mount Vernon Cancer Centre, UK). The study will open for recruitment in September 2022, and the estimated primary recruitment completion date is in September 2024 and study completion date in September 2026.

Patient and Public Involvement and Engagement (PPIE)

For peer review only

We have sought input from the Leeds Cancer PPIE team and Leeds Radiotherapy user group into the protocol design, lay summary and patient information leaflet and have acted on the information provided.

Consent and withdrawal

All participants will give written informed consent before entering the study and before any assessments or interventions related to the study are undertaken. Consent will be taken by the direct care clinical oncologist or a member of the RO-PIP research team e.g. clinical research fellow or research nurse. Optional consent will be sought for taking extra blood and urine samples for the translational study component. Participants are free to withdraw at any time, or at the discretion of the chief or principal investigator. In the event of withdrawal, any data collected up until that point will be kept and potentially included in any analyses.

Eligibility criteria

The inclusion criteria are: (i) Male individuals aged over 18 years; (ii) Histologically confirmed locally recurrent prostate cancer (following previous radiotherapy no less than 2 years ago); (iii) No metastatic disease; (iv) Able and willing to provide an informed consent to participate; (v) World Health Organisation (WHO) performance status 0-2; (vi) Reasonable urinary function (IPSS < 20 and Qmax > 10 ml/second on flow tests); (vii) Greater than 10 year life expectancy

The exclusion criteria are: (i) Patients who are unfit for a general anaesthetic due to other comorbidities; (ii) Clinical or radiological evidence of metastatic prostate disease; (iii) Any patient with a medical or psychiatric condition that impairs their ability to give informed consent. (iv) contraindication or intolerance of magnetic resonance scanning; (v) Prior prostatectomy; (vi) History of inflammatory bowel disease.

Assignment of interventions

Following confirmation of written consent and eligibility, participants will be randomised into the trial by the Leeds Clinical Trials Research Unit (CTRU). Patients will be randomised on a 1:1 basis to receive either HDR-BT or SBRT. Patients will be randomised using stratified permuted blocks, stratified by recruiting site and previous ADT therapy. Randomisation will be performed centrally using the CTRU 9-5 telephone randomisation system. Authorisation codes and personal identification numbers (PINs), provided by the CTRU, will be required to access the randomisation system

Interventions

For peer review only

HDR Brachytherapy

Two HDR-BT treatment schedules, either a single fraction 19Gy treatment or 27Gy in 2 fractions approximately 2 weeks apart will be used to be decided by treating centre.

Gross tumour volume (GTV) will be delineated based on the intra-prostatic lesion defined on the multi-parametric MRI with or without additional diagnostic PET-CT information; the clinical target volume (CTV) is generated by applying an isotropic 3mm margin constrained by the urethra (where applicable) and rectum. The CTV and planning target volume (PTV) are considered to be the same structures. The rectum, urethra and bladder should be contoured as organs at risk as per the ESTRO guidelines [23].

Stereotactic body radiation therapy

Patients will receive 5 fractions of 7.25Gy per fraction which will be delivered alternate days over no more than 2 weeks to provide a total dose of 36.25Gy. Radiotherapy may be delivered using CyberKnife, linear accelerator or MR-linear accelerator. Implanted prostate markers and SpaceOAR may be used as per centre standard of care.

Gross tumour volume (GTV) will be delineated based on the intra-prostatic lesion defined on the multiparametric MRI with or without additional diagnostic PET-CT information; a clinical target volume (CTV) will be delineated comprising either the whole prostate or for focal treatment the GTV with a 3mm margin constrained to the prostate boundaries. The CTV will then be grown by 3-5mm (dependent on departmental policy and image guidance technique) to generate a PTV. The rectum, bladder, bowel loops (where appropriate) and femoral heads will be contoured as organs at risk.

Additional Interventions

Androgen deprivation therapy (ADT) may be initiated at the discretion of the treating oncologist but this must be started by the time of the first salvage radiotherapy treatment (at first fraction of SBRT or at HDR-BT).

Toxicity Assessment

Clinician reported treatment toxicity will be summarised at each time point as the proportion of patients experiencing each toxicity, summarised by maximum grade experienced as per Common Terminology Criteria for Adverse Events (CTCAE) version 5.0.

For peer review only

PROMs Assessment

Changes in patient reported Health-Related Quality of life (HRQoL)/ PROMs will be assessed using the following validated questionnaires: EPIC-26 (prostate cancer related QoL and functional outcomes), EORTC QLQ-C30 (general QoL score) and IPSS (urinary and sexual functional outcomes). The specific time points for these evaluations are:

- Baseline assessment (prior to salvage treatment)
- 1 month post treatment completion
- 3 months post treatment completion
- 6 months post treatment completion
- 12 months post treatment completion
- 24 months post treatment completion

The PROMs and Quality of Life assessments will not require a separate face to face meeting as these will be posted out to the participants. Follow-up after 2 years will be according to local policy.

Translational MRI Assessment

All patients will have three multiparametric MRIs (including standard anatomical sequences and functional sequences) which will be paired with PROMs assessments at the same time points (at baseline and then post-treatment at 1 month and 1 year). The purpose of this imaging component is four fold:

- To optimise a multiparametric MRI scanning protocol across three institutions incorporating intravoxel incoherent motion (IVIM), dynamic contrast enhanced (DCE) imaging and blood oxygenation level dependent (BOLD) sequences
- To evaluate image quality and reproducibility of prostate functional imaging for detecting tissue perfusion and hypoxia
- To investigate prostate and pelvic anatomical and functional imaging changes related to prostate reirradiation and how this relates to patient reported toxicity side effects (determined by PROMs).
- To study the hypoxia MRI changes seen in the prostate in association with biopsy derived hypoxia-associated gene signature.

MRI scans done within the research study will be stored on the Leeds Teaching Hospitals Picture Archive and Communication System (PACS) server and on the local hospital PACS server where the images were obtained.

For peer review only

Translational Biological Assessment

The aim of this study component is to collect biological parameters that are prognostic and predictive markers of radiotherapy response and correlate this with imaging. From a biological stance the following sample collection will be relevant for assessing this:

- Tissue collection (prostate biopsy including original diagnostic and local recurrence sample) to measure the presence of a hypoxia-associated gene signature.
- Urine collection to measure the inflammatory response via damage-associated molecular patterns.
- Blood sample collection (20-30ml) to measures changes in cytokine response following reirradiation and other proteomics analyses.

The long-term storage arrangement for the research data arising from these biological samples will follow The University of Manchester Biobank (site of processing for biological samples) good practice for research guidance on clinical samples. Participants will have the option to consent to making their biological samples available for future research. The biological sample research data will be stored for 20 years once the study has ended.

Baseline and follow-up evaluation

Table 1 shows the full schedule of events.

Table 1: RO-PIP schedule of events.

Time-point / Item	* Baseline	During RT	1 month FU	3 month FU	6 month FU	12 month FU	18 month FU	24 month FU
Informed consent	x							
Registration	x							
Testosterone	x							
Confirmation of eligibility	x							
Randomisation to BT or SBRT	x							
Pre-treatment Prostate Biopsy sample requested and stored	x							
Collection of clinical history and outcome data	x							x
Radiotherapy (BT or SBRT) details	x	x						
PSA test	x			x	x	x	x	x
Functional prostate mpMRI	x		x			x		

For peer review

PROM assessment (EPIC-26, EORTC QLQ-C30 and IPSS)	x		x	x	x	x		x
Research blood test/ Urine	x		x	x	x			
Clinician reported adverse events		x	x	x	x	x	x	x

Key: FU=follow-up, RT=radiotherapy, BT=brachytherapy, EBRT= External Beam Radiotherapy, mpMRI=multi-parametric magnetic resonance imaging, PROM=patient recorded outcome measure, PSA=prostate specific antigen, EORTC QLQ = European Organization for the Research and Treatment of Cancer Quality of Life Questionnaire, EPIC-26= Expanded Prostate Cancer Index Composite-26, IPSS= international prostate symptom score

Outcomes

Primary outcome

1. Recruitment rates for the whole 24-month recruitment period will be reported overall and per recruiting site. The average recruitment rate per month and in total over the formal monitoring period will be reported.

The study recruitment period is 24 months. To show that patient recruitment targets for a phase III RCT can be met within an adequate timeframe, a “steady state” of recruitment should be observed. In this feasibility study, formal monitoring of recruitment will begin from the start of the patient recruitment where an average of two patients per month must be randomised over the remaining recruitment period in order to demonstrate a “steady state” of recruitment.

Secondary outcomes

1. Incidence of patient reported acute (0-3 months) and long-term toxicity (>3 months) and impact on QoL determined by EPIC-26 (prostate cancer related QoL and functional outcomes), EORTC QLQ-C30 (general QoL score) and international prostate symptom score (IPSS) (urinary and sexual functional outcomes) measurements (Key secondary endpoint).
2. Incidence of clinician-reported treatment toxicity as per Common Terminology Criteria for Adverse Events (CTCAE) version 5.0.
3. Other feasibility endpoints include screening log summaries, treatment and questionnaire compliance, withdrawal rate and reasons for non-recruitment.

Exploratory outcomes

1. MRI biomarkers at 1 month and 1 year post-treatment predictive of toxicity based on PROMs.

For peer review only

2. Hypoxia levels based on a hypoxia associated gene signature obtained from the pre-salvage RT biopsy correlated with MRI biomarkers.
3. Changes in the levels of inflammatory cytokine signatures from urine and blood obtained at baseline and after reirradiation in relation to PROMs.
4. Multiple measures of image quality and reproducibility of prostate functional imaging (e.g. diffusion coefficient values from IVIM sequences) for measuring tumour biology will be summarised.

Sample size

This is an exploratory feasibility study, and there is no informative data in the published literature on which to base a sample size calculation. Therefore, a formal statistical power calculation has not been performed. Feasibility studies are not usually sufficiently powered to provide estimates of effect size, but instead aim to determine the feasibility of specific study aspects and to enable estimation of sample size parameters to inform future studies. For this feasibility study, we plan to recruit 60 patients in total (i.e. 30 to each treatment arm) from three UK hospitals (Leeds Cancer Centre, The Christie and Mount Vernon Cancer Centre), all of which are high-volume tertiary prostate cancer treatment centres. This sample size has been informed by the National Institute of Health Research (NIHR) guidance on feasibility sample sizes and the toxicity outcome data from a recent systematic review[14,24]. Few studies have evaluated PROMs feasibility, establishing the need for this trial[25]. It is estimated that this number of participants will provide an adequate sample to estimate the toxicity rates for the key secondary endpoint in each arm (i.e. 30 per arm)[26,27]. Recruiting 60 patients over 2 years, across 3 centres, would mean an average recruitment rate of 1 patient per centre per month. This information, combined with the estimated toxicity rates from the study, will be used to determine feasibility of a subsequent larger scale RCT.

Recruitment

Sixty patients will be recruited over a 24-month period, approximately 2-3 patients per month (across 3 sites).

Reporting

No formal interim analysis will take place however a study report will be produced for review by the independent data monitoring and ethics committee (DMEC) approximately midway through the study. The aim of the report is to evaluate and monitor the key study objectives (i.e. recruitment rates, number of participants taking up their treatment allocation), as well as expected adverse events (AEs) and serious adverse events (SAEs) and the delivery time of HDR-BT or SBRT post-randomisation.

For peer review only - http://bmjopen.bmj.com/site/about/guidelines.xhtml

Data collection, management and analysis

Withdrawal of Participants

Participants who withdraw their consent to the study will be taken off the study. The research team will keep any tissue, blood, urine samples and imaging data already collected and continue analysis (unless the patient requests the destruction of samples and data). Patients will be consented from the outset to continue collecting follow-up data even if patients are withdrawn from the trial, cannot tolerate MRI scans, unable to continue treatment or do not complete all PROMs time points.

Data management

Study data will be managed by the trial coordinator and research fellow under the supervision of the chief investigator and the study statistician.

Data stored on hospital computers will be password protected and in locked rooms in the local hospital radiotherapy and/or radiology (imaging data) departments, only accessible by the local research team. Each patient is assigned a unique patient study ID number at enrolment (based on site and trial number allocated during randomisation step) which will be used on all trial documentation. This pseudoanonymisation step will prevent the patient from being identified by those outside the local research team. The local investigator will keep a subject enrolment and identification log that contains the key to the code, i.e. a record of the personal identification data linked to each patient study ID number.

In compliance with Good Clinical Practice guidelines and in accordance with the University of Leeds Code of Conduct and Research Ethics, the chief or local principal investigator will maintain all records regarding the conduct of the RO-PIP study. These will be securely archived for up to 20 years if required.

Statistical analysis

As this is a feasibility study, it will not involve hypothesis testing to identify whether the intervention has had an impact. Instead, data analysis will be descriptive and involve summary statistics. The analysis of all primary endpoint and all secondary endpoints relating to recruitment and withdrawals from the trial will take place at the end of the 24-month recruitment period. Final analysis of all other endpoint data will be carried out 6 months after the final participant has been randomised.

Future Work

For peer review only

Demonstrating feasibility will facilitate a larger randomised study comparing salvage re-irradiation options with ADT alone, the usual management option. This study would have primary endpoints of survival (overall and metastasis free). This is the first time that a hypoxia gene expression signature will be studied with hypoxia imaging in a prospective cohort of patients with radiorecurrent prostate cancer and could lead to the further studies investigating the introduction in clinical practice of tumour hypoxia testing (from biopsy and/or imaging) and the biological individualisation of radiotherapy. Given the large number of prostate cancer patients who undergo radiotherapy each year, this would have a significant impact on personalised medicine in the UK.

Trial oversight

A trial management group will be convened for the study, consisting of the chief investigator, principal investigators (for each site), research fellow, trial administrator, and research nurse. The group will meet monthly. The study sponsor (University of Leeds) will monitor the conduct of the trial. A trial report will be produced by the DMEC midway through the study

Ethics statements

This study has been approved by the Yorkshire & The Humber - Bradford Leeds Research Ethics Committee (Reference: 21/YH/0305, IRAS: 297060, January 2022).

Dissemination

The results will be presented in national and international conferences, published in peer-reviewed journals, publicised via social media channels such as twitter, and will be communicated to relevant stakeholders. A plain English report will be shared with the study participants, patients' organisations, PPIE groups and media.

Patient consent for publication

Not applicable.

For peer review only

References

1. Cancer Research UK. Cancer Incidence from Cancer Intelligence Statistical Information Team at Cancer Research UK [Internet]. 2019.
2. Bray F, Ferlay J, Soerjomataram I, Siegel RL, Torre LA, Jemal A. Global cancer statistics 2018: GLOBOCAN estimates of incidence and mortality worldwide for 36 cancers in 185 countries. CA Cancer J Clin [Internet]. 2018;68:394–424.
3. Mottet N, Bellmunt J, Bolla M, Briers E, Cumberbatch MG, De Santis M, et al. EAU-ESTRO-SIOG Guidelines on Prostate Cancer. Part 1: Screening, Diagnosis, and Local Treatment with Curative Intent. Eur Urol [Internet]. 2017;71(4):618–29.
4. Cancer Research UK . Prostate cancer treatment statistics [Internet]. 2017.
5. Beckendorf V, Guerif S, Le Prisé E, Cosset JM, Bougnoux A, Chauvet B, et al. 70 Gy versus 80 Gy in localized prostate cancer: 5-year results of GETUG 06 randomized trial. Int J Radiat Oncol Biol Phys [Internet]. 2011;80(4):1056–63.
6. Zumsteg ZS, Spratt DE, Romesser PB, Pei X, Zhang Z, Kollmeier M, et al. Anatomical patterns of recurrence following biochemical relapse in the dose escalation era of external beam radiotherapy for prostate cancer. J Urol [Internet]. 2015;194(6):1624-1630.
7. Ma TM, Chu F-I, Sandler H, Feng FY, Efstathiou JA, Jones CU, et al. Local Failure Events in Prostate Cancer Treated with Radiotherapy: A Pooled Analysis of 18 Randomized Trials from the Meta-analysis of Randomized Trials in Cancer of the Prostate Consortium (LEVIATHAN). Eur Urol. 2022;
8. Jones CU, Hunt D, McGowan DG, Amin MB, Chetner MP, Bruner DW, et al. Radiotherapy and Short-Term Androgen Deprivation for Localized Prostate Cancer. N Engl J Med. 2011;365(2):107–18.
9. Cooperberg MR, Vickers AJ, Broering JM, Carroll PR. Comparative risk-adjusted mortality outcomes after primary surgery, radiotherapy, or androgen-deprivation therapy for localized prostate cancer. Cancer [Internet]. 2010;116:5226–5234.
10. Mbeutcha A, Chauveinc L, Bondiau PY, Chand ME, Durand M, Chevallier D, et al. Salvage prostate re-irradiation using high-dose-rate brachytherapy or focal stereotactic body radiotherapy for local recurrence after definitive radiation therapy. Radiat Oncol [Internet]. 2017;
11. Ingrosso G, Becherini C, Lancia A, Caini S, Ost P, Francolini G, et al. Nonsurgical Salvage Local Therapies for Radiorecurrent Prostate Cancer: A Systematic Review and Meta-analysis. Eur Urol Oncol [Internet]. 2019;3(2):183–97.
12. Chade DC, Eastham J, Graefen M, Hu JC, Karnes RJ, Klotz L, et al. Cancer control and functional outcomes of salvage radical prostatectomy for radiation-recurrent prostate cancer: A systematic review of the literature. Eur Urol [Internet].

For peer review only

2012;61(5):961–71.

13. Valle LF, Lehrer EJ, Markovic D, Elashoff D, Levin-Epstein R, Karnes RJ, et al. A Systematic Review and Meta-analysis of Local Salvage Therapies After Radiotherapy for Prostate Cancer (MASTER). Eur Urol. 2020;1(310):1–13.
14. Zhong J, Slevin F, Scarsbrook AF, Serra M, Choudhury A, Hoskin PJ, et al. Salvage Reirradiation Options for Locally Recurrent Prostate Cancer: A Systematic Review. Front Oncol. 2021;11.
15. Chatzikonstantinou G, Zamboglou N, Rödel C, Zoga E, Strouthos I, Butt SA, et al. High-dose-rate brachytherapy as salvage modality for locally recurrent prostate cancer after definitive radiotherapy: Asystematic review. Strahlentherapie und Onkol [Internet]. 2017;193:683–691.
16. Chitmanee P, Tsang Y, Tharmalingam H, Hamada M, Alonzi R, Ostler P, et al. Single-Dose Focal Salvage High Dose Rate Brachytherapy for Locally Recurrent Prostate Cancer. Clin Oncol [Internet]. 2020;32(4):259-265.
17. Murgic J, Morton G, Loblaw A, D'Alimonte L, Ravi A, Wronski M, et al. Focal Salvage High Dose-Rate Brachytherapy for Locally Recurrent Prostate Cancer After Primary Radiation Therapy Failure: Results From a Prospective Clinical Trial. Int J Radiat Oncol Biol Phys [Internet]. 2018;102(3):561-567.
18. Slevin F, Hodgson S, Rodda SL, Bownes P, Bottomley D, Adiotomre E, et al. Efficacy and toxicity outcomes for patients treated with focal salvage high dose rate brachytherapy for locally recurrent prostate cancer. Clin Transl Radiat Oncol [Internet]. 2020;23:20-26.
19. Hathout L, Mahmoud O, Wang Y, Vergalasova I, Barkati M, Després P, et al. A Phase 2 Randomized Pilot Study Comparing High-Dose-Rate Brachytherapy and Low-Dose-Rate Brachytherapy as Monotherapy in Localized Prostate Cancer. Adv Radiat Oncol [Internet]. 2019;4(4):631-640.
20. Zilli T, Benz E, Dipasquale G, Rouzaud M, Miralbell R. Reirradiation of Prostate Cancer Local Failures After Previous Curative Radiation Therapy: Long-Term Outcome and Tolerance. Int J Radiat Oncol Biol Phys [Internet]. 2016;96:218–322.
21. Pasquier D, Martinage G, Janoray G, Rojas DP, Zerini D, Goupy F, et al. Salvage Stereotactic Body Radiation Therapy for Local Prostate Cancer Recurrence After Radiation Therapy: A Retrospective Multicenter Study of the GETUG. Int J Radiat Oncol Biol Phys [Internet]. 2019;105(4):727-734.
22. Chan A-W, Tetzlaff JM, Altman DG, Laupacis A, Gøtzsche PC, Krleža-Jerić K, et al. SPIRIT 2013 Statement: Defining Standard Protocol Items for Clinical Trials. Ann Intern Med. 2013;158(3):200.
23. Salembier C, Villeirs G, De Bari B, Hoskin P, Pieters BR, Van Vulpen M, et al. ESTRO

For peer review only

ACROP consensus guideline on CT- and MRI-based target volume delineation for primary radiation therapy of localized prostate cancer. Radiother Oncol. 2018;127(1):49–61.

24. Hooper R. Justifying sample size for a feasibility study (NIHR Guidance) [Internet]. 2021.

25. Skolarus TA, Dunn RL, Sanda MG, Chang P, Greenfield TK, Litwin MS, et al. Minimally important difference for the expanded prostate cancer index composite short form. Urology [Internet]. 2015;85(1):101–106.

26. Lancaster GA, Dodd S, Williamson PR. Design and analysis of pilot studies: recommendations for good practice. J Eval Clin Pract. 2004;10(2):307–12.

27. Billingham SA, Whitehead AL, Julious SA. An audit of sample sizes for pilot and feasibility trials being undertaken in the United Kingdom registered in the United Kingdom Clinical Research Network database. BMC Med Res Methodol. 2013;13(1):104.

For peer review only

Figure Legends

Figure 1: Schematic overview of study.

Key – CT = Computed Tomography, PET = positron emission tomography, mpMRI = multi-parametric magnetic resonance imaging, EORTC QLQ = European Organization for the Research and Treatment of Cancer Quality of Life Questionnaire, IPSS = international prostate symptom score, EPIC = Expanded Prostate Cancer Index Composite, QoL = quality of life, CTCAE = Common Terminology Criteria for Adverse Events.

Footnotes

Author Statement/ Contributors:

JZ, AC, PH, AH, SB, PB and CT developed the protocol, and co-wrote the ethics application for the study. All authors contributed to the study design and protocol. JZ, DB, MD, DM and AS contributed to the development of the MR imaging aspects. JZ, KR, RR, AC, PH, CW and AH developed the translational biology parts of the study. All authors contributed to the writing, critical review and revisions of the manuscript.

Funding:

This work is supported by a CRUK award: grant number 95653118.

Competing interests:

None declared.

Data Availability Statement

Data sharing not applicable as no datasets generated and/or analysed for this study yet.

For peer review only

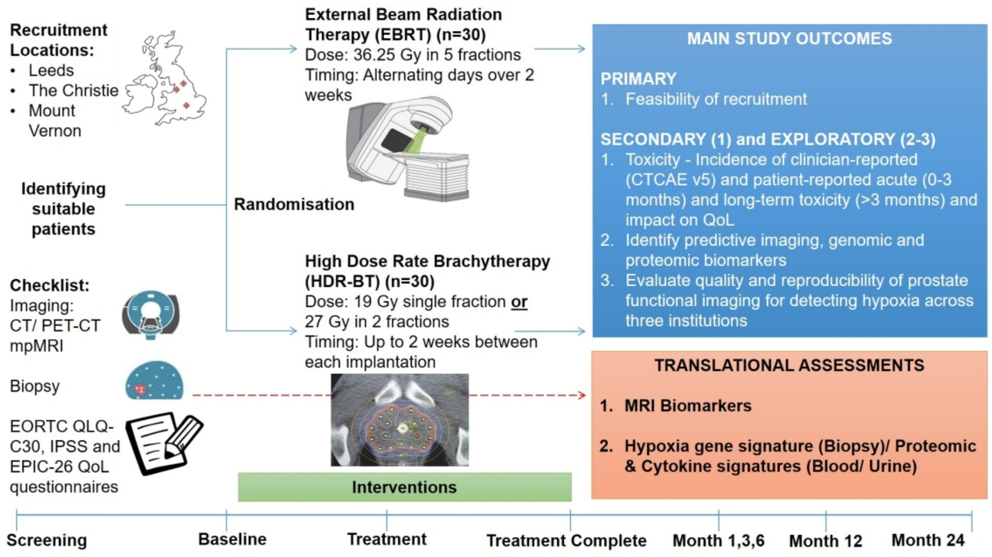

Figure 1: Schematic overview of study.
Key – CT = Computed Tomography, PET = positron emission tomography, mpMRI = multi-parametric magnetic resonance imaging, EORTC QLQ = European Organization for the Research and Treatment of Cancer Quality of Life Questionnaire, IPSS = international prostate symptom score, EPIC = Expanded Prostate Cancer Index Composite, QoL = quality of life, CTCAE = Common Terminology Criteria for Adverse Events.

336x189mm (150 x 150 DPI)

SPIRIT 2013 Checklist: Recommended items to address in a clinical trial protocol and related documents*

Section/item	ItemNo	Page No	Description
			**Administrative information**
Title	1	1	Descriptive title identifying the study design, population, interventions, and, if applicable, trial acronym
Trial registration	2a	2	Trial identifier and registry name. If not yet registered, name of intended registry
	2b	2	All items from the World Health Organization Trial Registration Data Set
Protocol version	3	1	Date and version identifier
Funding	4		Sources and types of financial, material, and other support
Roles and responsibilities	5a	1	Names, affiliations, and roles of protocol contributors
	5b	1	Name and contact information for the trial sponsor
	5c	1	Role of study sponsor and funders, if any, in study design; collection, management, analysis, and interpretation of data; writing of the report; and the decision to submit the report for publication, including whether they will have ultimate authority over any of these activities
	5d	1	Composition, roles, and responsibilities of the coordinating centre, steering committee, endpoint adjudication committee, data management team, and other individuals or groups overseeing the trial, if applicable (see Item 21a for data monitoring committee)
**Introduction**			
Background and rationale	6a	4	Description of research question and justification for undertaking the trial, including summary of relevant studies (published and unpublished) examining benefits and harms for each intervention

For peer review only

	6b	4	Explanation for choice of comparators
Objectives	7	2	Specific objectives or hypotheses
Trial design	8	2	Description of trial design including type of trial (eg, parallel group, crossover, factorial, single group), allocation ratio, and framework (eg, superiority, equivalence, noninferiority, exploratory)
			**Methods: Participants, interventions, and outcomes**
Study setting	9	6	Description of study settings (eg, community clinic, academic hospital) and list of countries where data will be collected. Reference to where list of study sites can be obtained
Eligibility criteria	10	6	Inclusion and exclusion criteria for participants. If applicable, eligibility criteria for study centres and individuals who will perform the interventions (eg, surgeons, psychotherapists)
Interventions	11a	7	Interventions for each group with sufficient detail to allow replication, including how and when they will be administered
	11b	6	Criteria for discontinuing or modifying allocated interventions for a given trial participant (eg, drug dose change in response to harms, participant request, or improving/worsening disease)
	11c	7	Strategies to improve adherence to intervention protocols, and any procedures for monitoring adherence (eg, drug tablet return, laboratory tests)
	11d	7	Relevant concomitant care and interventions that are permitted or prohibited during the trial
Outcomes	12	10/11	Primary, secondary, and other outcomes, including the specific measurement variable (eg, systolic blood pressure), analysis metric (eg, change from baseline, final value, time to event), method of aggregation (eg, median, proportion), and time point for each outcome. Explanation of the clinical relevance of chosen efficacy and harm outcomes is strongly recommended
Participant timeline	13	9/10	Time schedule of enrolment, interventions (including any run-ins and washouts), assessments, and visits for participants. A schematic diagram is highly recommended (see Figure)

For peer review only - http://bmjopen.bmj.com/site/about/guidelines.xhtml

Sample size	14	12	Estimated number of participants needed to achieve study objectives and how it was determined, including clinical and statistical assumptions supporting any sample size calculations
Recruitment	15	12	Strategies for achieving adequate participant enrolment to reach target sample size
			**Methods: Assignment of interventions (for controlled trials)**
Allocation:			
Sequence generation	16a	12	Method of generating the allocation sequence (eg, computer-generated random numbers), and list of any factors for stratification. To reduce predictability of a random sequence, details of any planned restriction (eg, blocking) should be provided in a separate document that is unavailable to those who enrol participants or assign interventions
Allocation concealment mechanism	16b	12	Mechanism of implementing the allocation sequence (eg, central telephone; sequentially numbered, opaque, sealed envelopes), describing any steps to conceal the sequence until interventions are assigned
Implementation	16c	12	Who will generate the allocation sequence, who will enrol participants, and who will assign participants to interventions
Blinding (masking)	17a	n/a	Who will be blinded after assignment to interventions (eg, trial participants, care providers, outcome assessors, data analysts), and how
	17b	n/a	If blinded, circumstances under which unblinding is permissible, and procedure for revealing a participant's allocated intervention during the trial
			**Methods: Data collection, management, and analysis**

For peer review only - http://bmjopen.bmj.com/site/about/guidelines.xhtml

Data collection methods	18a	12/13	Plans for assessment and collection of outcome, baseline, and other trial data, including any related processes to promote data quality (eg, duplicate measurements, training of assessors) and a description of study instruments (eg, questionnaires, laboratory tests) along with their reliability and validity, if known. Reference to where data collection forms can be found, if not in the protocol
	18b	13	Plans to promote participant retention and complete follow-up, including list of any outcome data to be collected for participants who discontinue or deviate from intervention protocols
Data management	19	13	Plans for data entry, coding, security, and storage, including any related processes to promote data quality (eg, double data entry; range checks for data values). Reference to where details of data management procedures can be found, if not in the protocol
Statistical methods	20a	13	Statistical methods for analysing primary and secondary outcomes. Reference to where other details of the statistical analysis plan can be found, if not in the protocol
	20b	14	Methods for any additional analyses (eg, subgroup and adjusted analyses)
	20c	13/14	Definition of analysis population relating to protocol non-adherence (eg, as randomised analysis), and any statistical methods to handle missing data (eg, multiple imputation)
			**Methods: Monitoring**
Data monitoring	21a	13	Composition of data monitoring committee (DMC); summary of its role and reporting structure; statement of whether it is independent from the sponsor and competing interests; and reference to where further details about its charter can be found, if not in the protocol. Alternatively, an explanation of why a DMC is not needed
	21b	12	Description of any interim analyses and stopping guidelines, including who will have access to these interim results and make the final decision to terminate the trial

Harms	22	12	Plans for collecting, assessing, reporting, and managing solicited and spontaneously reported adverse events and other unintended effects of trial interventions or trial conduct
Auditing	23	12	Frequency and procedures for auditing trial conduct, if any, and whether the process will be independent from investigators and the sponsor
			**Ethics and dissemination**
Research ethics approval	24	2	Plans for seeking research ethics committee/institutional review board (REC/IRB) approval
Protocol amendments	25	12	Plans for communicating important protocol modifications (eg, changes to eligibility criteria, outcomes, analyses) to relevant parties (eg, investigators, REC/IRBs, trial participants, trial registries, journals, regulators)
Consent or assent	26a	6	Who will obtain informed consent or assent from potential trial participants or authorised surrogates, and how (see Item 32)
	26b	6	Additional consent provisions for collection and use of participant data and biological specimens in ancillary studies, if applicable
Confidentiality	27	13	How personal information about potential and enrolled participants will be collected, shared, and maintained in order to protect confidentiality before, during, and after the trial
Declaration of interests	28	18	Financial and other competing interests for principal investigators for the overall trial and each study site
Access to data	29	14	Statement of who will have access to the final trial dataset, and disclosure of contractual agreements that limit such access for investigators
Ancillary and post-trial care	30	19	Provisions, if any, for ancillary and post-trial care, and for compensation to those who suffer harm from trial participation

For peer review only

Dissemination policy	31a	15	Plans for investigators and sponsor to communicate trial results to participants, healthcare professionals, the public, and other relevant groups (eg, via publication, reporting in results databases, or other data sharing arrangements), including any publication restrictions
	31b	15	Authorship eligibility guidelines and any intended use of professional writers
	31c	15	Plans, if any, for granting public access to the full protocol, participant-level dataset, and statistical code
**Appendices**			
Informed consent materials	32	Available	Model consent form and other related documentation given to participants and authorised surrogates
Biological specimens	33	14	Plans for collection, laboratory evaluation, and storage of biological specimens for genetic or molecular analysis in the current trial and for future use in ancillary studies, if applicable

*It is strongly recommended that this checklist be read in conjunction with the SPIRIT 2013 Explanation & Elaboration for important clarification on the items. Amendments to the protocol should be tracked and dated. The SPIRIT checklist is copyrighted by the SPIRIT Group under the Creative Commons "Attribution-NonCommercial-NoDerivs 3.0 Unported" license.

For peer review only

BMJ Open

Reirradiation Options for Previously Irradiated Prostate cancer (RO-PIP): Feasibility study investigating toxicity outcomes following reirradiation with stereotactic body radiotherapy (SBRT) vs. high dose-rate brachytherapy (HDR-BT)

Journal:	*BMJ Open*
Manuscript ID	bmjopen-2022-068580.R1
Article Type:	Protocol
Date Submitted by the Author:	07-Oct-2022
Complete List of Authors:	Zhong, Jim; University of Leeds; St James's University Hospital, Diagnostic and Interventional Radiology<br>Brown, Sarah; University of Leeds, Clinical Trials Research Unit<br>Serra, Maria; The Christie NHS Foundation Trust, Department of Clinical Oncology<br>Shuttleworth, Pam; St James's University Hospital, Department of Clinical Oncology<br>Bownes, Peter; St James's University Hospital, Department of Clinical Oncology<br>Thompson, Christopher; St James's University Hospital, Department of Clinical Oncology<br>Reed, Rachel; The University of Manchester, Division of Cancer Sciences<br>Reeves, Kimberley; The University of Manchester, Division of Cancer Sciences<br>Dubec, Michael; The University of Manchester, Division of Cancer Sciences; The Christie NHS Foundation Trust, Department of Clinical Oncology<br>McHugh, Damien; The University of Manchester, Division of Cancer Sciences; The Christie NHS Foundation Trust, Department of Clinical Oncology<br>Eccles, Cynthia; The Christie NHS Foundation Trust, Department of Clinical Oncology; The University of Manchester<br>Chuter, Robert; The Christie NHS Foundation Trust, Department of Clinical Oncology; The University of Manchester, Division of Cancer Sciences<br>Tsang, Yat Man; Mount Vernon Cancer Centre, Department of Clinical Oncology<br>Taylor, N. Jane; Mount Vernon Hospital, Paul Strickland Scanner Centre<br>West, Catharine ; The University of Manchester Division of Cancer Sciences<br>Buckley, David; University of Leeds, Leeds Institute of Cardiovascular and Metabolic Medicine<br>Scarsbrook, Andrew; Leeds Teaching Hospitals NHS Trust, Department of Radiology; University of Leeds, Leeds Institute of Medical Research<br>Choudhury, Ananya; The Christie NHS Foundation Trust, Department of Clinical Oncology; The University of Manchester, Division of Cancer Sciences

	Hoskin, Peter; Mount Vernon Cancer Centre, Department of Clinical Oncology; The University of Manchester, Division of Cancer Sciences Henry, Ann; University of Leeds, Leeds Institute of Medical Research; St James's University Hospital, Department of Clinical Oncology
**Primary Subject Heading**:	Oncology
Secondary Subject Heading:	Radiology and imaging
Keywords:	Prostate disease < UROLOGY, RADIOTHERAPY, Magnetic resonance imaging < RADIOLOGY & IMAGING

Reirradiation Options for Previously Irradiated Prostate cancer (RO-PIP): Feasibility study investigating toxicity outcomes following reirradiation with stereotactic body radiotherapy (SBRT) vs. high dose-rate brachytherapy (HDR-BT)

*Jim Zhong[1,2], Sarah Brown[3], Maria Serra[4], Pam Shuttleworth[2], Peter Bownes[2], Christopher Thompson[2], Rachel Reed[5], Kimberley Reeves[5], Michael Dubec[4,5], Damien McHugh[4,5], Cynthia Eccles[4,5], Robert Chuter[4,5], Yat Man Tsang[6], N. Jane Taylor[7], Catharine West[5], David L. Buckley[8], Andrew Scarsbrook[1,2], Ananya Choudhury[4,5], Peter Hoskin[4,5,6], Ann Henry[1,2]

1. Leeds Institute of Medical Research, University of Leeds, Leeds, UK
2. Leeds Cancer Centre, St James's University Hospital, Leeds Teaching Hospitals National Health Service (NHS) Trust, Beckett Street, Leeds, LS9 7TF
3. Leeds Institute of Clinical Trials Research (LICTR), University of Leeds, Leeds, UK
4. The Christie National Health Service (NHS) Foundation Trust, Manchester, UK
5. Division of Cancer Sciences, School of Medical Sciences, Faculty of Biology, Medicine and Health, The University of Manchester, The Christie Hospital, Manchester, UK
6. Mount Vernon Cancer Centre, Northwood, UK
7. Paul Strickland Scanner Centre, Mount Vernon Hospital, Northwood, Middlesex HA6 2RN
8. Leeds Institute of Cardiovascular and Metabolic Medicine, University of Leeds, Leeds, UK

*Corresponding author details

Dr Jim Zhong

Clinical Research Fellow and Radiology Registrar

Leeds Institute of Medical Research

St. James's University Hospital

University of Leeds

Beckett Street

Leeds

LS9 7TF

UK

Email: jim.zhong@nhs.net

For peer review only

Word count (excluding title, abstract and references) = 3267 (MAX 4000)

Acknowledgements: AC, PH, CE, CW are supported by the NIHR Manchester Biomedical Research Centre, UK. AS is supported by the Cancer Research UK (CRUK) Leeds Radiotherapy Research Centre of Excellence (RadNet C19942/A28832). JZ is supported by a CRUK Award (Leeds-Manchester Stella Erdheim Clinical PhD Fellowship – Grant Reference Number 95653117/95653118). This work was also supported by the Cancer Research UK Manchester Centre award [CTRQQR-2021\100010].

For peer review only

Abstract

Introduction

Radiotherapy is the most common curative treatment for non-metastatic prostate cancer, however up to 13% of patients will develop local recurrence within 10 years. Patients can undergo further and potentially curative treatment including salvage surgery, brachytherapy (BT), external beam radiotherapy (EBRT), high intensity focused ultrasound and cryotherapy. Systematic review shows that high dose rate (HDR) BT and stereotactic body radiotherapy (SBRT) have the best outcomes in terms of biochemical control and lowest side effects. The RO-PIP trial aims to determine the feasibility of recruitment to a trial randomising patients to salvage HDR-BT or SBRT and provide prospective data on patient recorded toxicity outcomes that will inform a future phase III trial.

Methods and analysis

The primary endpoint of the RO-PIP feasibility study is to evaluate the patient recruitment potential over 2 years to a trial randomising to either SBRT or HDR-BT for patients who develop local recurrence of prostate cancer following previous radiation therapy. The aim is to recruit 60 patients across 3 sites over 2 years and randomise 1:1 to SBRT or HDR-BT. Secondary objectives include recording clinician and patient reported outcome measures (PROMs) to evaluate treatment-related toxicity. In addition, the study aims to identify potential imaging, genomic and proteomic biomarkers that are predictive of toxicity and outcome based on hypoxia status, a prognostic marker of prostate cancer.

Ethics and dissemination

This study has been approved by the Yorkshire & The Humber - Bradford Leeds Research Ethics Committee (Reference: 21/YH/0305, IRAS: 297060, January 2022). The results will be presented in national and international conferences, published in peer-reviewed journals and will be communicated to relevant stakeholders. A plain English report will be shared with the study participants, patients' organisations and media. Trial registration number: ISRCTN 12238218

For peer review only

Article Summary

Strengths and limitations

- The RO-PIP study is the first to assess the feasibility to randomise men to different salvage radiation treatments for radio-recurrent prostate cancer.
- Randomised radiation treatment allocation will prevent selection bias
- Using patient reported outcome measures to collect treatment toxicity data will allow for more patient-centred care.
- The addition of translational components into study will reveal information on the systemic and local effect of prostate reirradiation and identify candidate radioresponse biomarkers for inclusion in a phase III trial.
- Due to the feasibility design of the study, it is not powered to assess for differences in outcome between SBRT and HDR-BT.

Keywords

Prostate cancer; radiotherapy

For peer review only

Introduction

Prostate cancer is the most common cancer in males in the United Kingdom with approximately 48,500 new diagnoses every year and this has increased over the last 10 years [1]. Worldwide, prostate cancer accounts for over 1.2 million new cases and causes over 350,000 deaths (3.8% of all deaths caused by cancer in men) annually [2]. Radiation therapy (RT) is the most common curative treatment for non-metastatic prostate cancer [3,4]. Despite advances in diagnostic imaging, RT delivery techniques and dose-escalation strategies, treatment failure remains common [5–7]. At 10-years, the biochemical failure rate following treatment for localised prostate cancer with RT alone is 41% [8]. Following dose-escalated RT, the most common site of cancer recurrence is in the prostate with 11-year cumulative incidence of local recurrence 7.2%, and 13% in intermediate, and high risk National Comprehensive Cancer Network (NCCN) prostate cancer groups respectively [7].

Multiple salvage options are available for locally recurrent disease including prostatectomy, reirradiation (with brachytherapy (BT) or external beam radiotherapy (EBRT)) and other focal therapies such as high-intensity focused ultrasound (HIFU) and cryotherapy (CRYO). The evidence on the long-term effectiveness and quality of life impact for these treatments are limited, however reirradiation techniques are the safest and most effective out of the currently available salvage treatment options [9–13]. Most of the published literature describes retrospective case-series with heterogeneous methodologies and radiation treatment techniques with no high level evidence for the superiority of any of the re-irradiation approaches or prospective comparative studies. Only a small proportion of patients with locally recurrent prostate cancer following primary radiotherapy (15-20%) undergo local salvage therapy according to the Cancer of the Prostate Strategic Urologic Research Endeavor (CaPSURE) registry [14].

Prostate BT involves the placement of sealed radiation sources into the prostate and offers the ability to deliver highly conformal high dose radiation with a steep dose gradient and rapid fall off in dose which minimises radiation toxicity to surrounding organs at risk, specifically the rectum and bladder [15]. Advances in image-guided targeted BT may allow for more precise and focused treatments [16–18]. BT can be delivered either via a permanent low-dose rate seed implant (LDR-BT), or via high-dose rate BT (HDR-BT) which uses a high-activity radiation source (e.g. iridium-192) that is temporarily introduced into applicators that are placed within the prostate, typically over 1-3 fractions. HDR-BT is less susceptible to issues related to prostate oedema and seed migration that might complicate dosimetry following LDR-BT. Current evidence suggests that HDR-BT affords lower toxicity, increased tolerability with similar oncological control compared to LDR-BT [19].

For peer review only

Previously, salvage EBRT techniques have been associated with higher rates of severe late toxicities and also poor local control [20]. Stereotactic body radiation therapy (SBRT), involving the delivery of a high dose of radiation to a highly conformal target volume with a steep dose gradient in a small number of fractions may have benefits. Potential advantages include increased sparing of normal tissues than other types of EBRT, and being less invasive compared to BT [21]. BT is also highly specialised and only available in specialist centres.

A prospective trial is required to describe the toxicity profiles for these two most promising options, HDR-BT and SBRT, to allow clinicians and patients to make an informed decision on the most appropriate salvage treatment choice and help inform a larger study with an efficacy endpoint. Strategies to personalise salvage treatment through finding predictive genomic and imaging biomarkers are also required to optimise treatment outcomes.
The RO-PIP trial is the first prospective randomised trial to determine the feasibility of recruitment to a trial comparing SBRT and HDR-BT for locally recurrent prostate cancer and inform power calculations for a definitive RCT. In addition, this feasibility trial will also quantify the impact on patient reported outcome measures (PROMs), quantify longitudinal functional MRI changes and assess proteomic, immune and genomic biomarkers.

Method

Study Design

The Standard Protocol Items for Randomized Trials (SPIRIT) checklist was adhered to when drafting the RO-PIP protocol[22]. A completed SPIRIT checklist can be found with the trial protocol submission. A schematic overview of the study is shown in Figure 1.

Study Setting

The planned study is a prospective two arm (HDR-BT and SBRT) randomised (1:1) feasibility trial aiming to recruit a total of 60 patients with locally recurrent prostate cancer across three tertiary referral oncology sites (Christie Hospital NHS Foundation Trust, Leeds Teaching Hospitals NHS Trust and Mount Vernon Cancer Centre, UK). The study will open for recruitment in September 2022, and the estimated primary recruitment completion date is in September 2024 and study completion date in September 2026.

Patient and Public Involvement and Engagement (PPIE)

For peer review only

We have sought input from the Leeds Cancer PPIE team and Leeds Radiotherapy user group into the protocol design, lay summary and patient information leaflet and have acted on the information provided.

Consent and withdrawal

All participants will give written informed consent before entering the study and before any assessments or interventions related to the study are undertaken. Consent will be taken by the direct care clinical oncologist or a member of the RO-PIP research team e.g. clinical research fellow or research nurse. Optional consent will be sought for taking extra blood and urine samples for the translational study component. Participants are free to withdraw at any time, or at the discretion of the chief or principal investigator. In the event of withdrawal, any data collected up until that point will be kept and potentially included in any analyses.

Eligibility criteria

The inclusion criteria are: (i) Male individuals aged over 18 years; (ii) Histologically confirmed locally recurrent prostate cancer (following previous radiotherapy no less than 2 years ago); (iii) No metastatic disease; (iv) Able and willing to provide an informed consent to participate; (v) World Health Organisation (WHO) performance status 0-2; (vi) Reasonable urinary function (IPSS < 20 and Qmax > 10 ml/second on flow tests); (vii) Greater than 10 year life expectancy

The exclusion criteria are: (i) Patients who are unfit for a general anaesthetic due to other comorbidities; (ii) Clinical or radiological evidence of metastatic prostate disease; (iii) Any patient with a medical or psychiatric condition that impairs their ability to give informed consent. (iv) contraindication or intolerance of magnetic resonance scanning; (v) Prior prostatectomy; (vi) History of inflammatory bowel disease.

Assignment of interventions

Following confirmation of written consent and eligibility, participants will be randomised into the trial by the Leeds Clinical Trials Research Unit (CTRU). Patients will be randomised on a 1:1 basis to receive either HDR-BT or SBRT. Patients will be randomised using stratified permuted blocks, stratified by recruiting site and previous ADT therapy. Randomisation will be performed centrally using the CTRU 9-5 telephone randomisation system. Authorisation codes and personal identification numbers (PINs), provided by the CTRU, will be required to access the randomisation system

Interventions

For peer review only

HDR Brachytherapy

Two HDR-BT treatment schedules, either a single fraction 19Gy treatment or 27Gy in 2 fractions approximately 2 weeks apart will be used to be decided by treating centre.

Gross tumour volume (GTV) will be delineated based on the intra-prostatic lesion defined on the multi-parametric MRI with or without additional diagnostic PET-CT information; the clinical target volume (CTV) is generated by applying an isotropic 3mm margin constrained by the urethra (where applicable) and rectum. The CTV and planning target volume (PTV) are considered to be the same structures. The rectum, urethra and bladder should be contoured as organs at risk as per the ESTRO guidelines [23].

Stereotactic body radiation therapy

Patients will receive 5 fractions of 7.25Gy per fraction which will be delivered alternate days over no more than 2 weeks to provide a total dose of 36.25Gy. Radiotherapy may be delivered using CyberKnife, linear accelerator or MR-linear accelerator. Implanted prostate markers and SpaceOAR may be used as per centre standard of care.

Gross tumour volume (GTV) will be delineated based on the intra-prostatic lesion defined on the multiparametric MRI with or without additional diagnostic PET-CT information; a clinical target volume (CTV) will be delineated comprising either the whole prostate or for focal treatment the GTV with a 3mm margin constrained to the prostate boundaries. The CTV will then be grown by 3-5mm (dependent on departmental policy and image guidance technique) to generate a PTV. The rectum, bladder, bowel loops (where appropriate) and femoral heads will be contoured as organs at risk.

Additional Interventions

Androgen deprivation therapy (ADT) may be initiated at the discretion of the treating oncologist but this must be started by the time of the first salvage radiotherapy treatment (at first fraction of SBRT or at HDR-BT).

Toxicity Assessment

Clinician reported treatment toxicity will be summarised at each time point as the proportion of patients experiencing each toxicity, summarised by maximum grade experienced as per Common Terminology Criteria for Adverse Events (CTCAE) version 5.0.

For peer review only

PROMs Assessment

Changes in patient reported Health-Related Quality of life (HRQoL)/ PROMs will be assessed using the following validated questionnaires: EPIC-26 (prostate cancer related QoL and functional outcomes), EORTC QLQ-C30 (general QoL score) and IPSS (urinary and sexual functional outcomes). The specific time points for these evaluations are:

- Baseline assessment (prior to salvage treatment)
- 1 month post treatment completion
- 3 months post treatment completion
- 6 months post treatment completion
- 12 months post treatment completion
- 24 months post treatment completion

The PROMs and Quality of Life assessments will not require a separate face to face meeting as these will be posted out to the participants. Follow-up after 2 years will be according to local policy.

Translational MRI Assessment

All patients will have three multiparametric MRIs (including standard anatomical sequences and functional sequences) which will be paired with PROMs assessments at the same time points (at baseline and then post-treatment at 1 month and 1 year). The purpose of this imaging component is four fold:

- To optimise a multiparametric MRI scanning protocol across three institutions incorporating intravoxel incoherent motion (IVIM), dynamic contrast enhanced (DCE) imaging and blood oxygenation level dependent (BOLD) sequences
- To evaluate image quality and reproducibility of prostate functional imaging for detecting tissue perfusion and hypoxia
- To investigate prostate and pelvic anatomical and functional imaging changes related to prostate reirradiation and how this relates to patient reported toxicity side effects (determined by PROMs).
- To study the hypoxia MRI changes seen in the prostate in association with biopsy derived hypoxia-associated gene signature.

MRI scans done within the research study will be stored on the Leeds Teaching Hospitals Picture Archive and Communication System (PACS) server and on the local hospital PACS server where the images were obtained.

For peer review only

Translational Biological Assessment

The aim of this study component is to collect biological parameters that are prognostic and predictive markers of radiotherapy response and correlate this with imaging. From a biological stance the following sample collection will be relevant for assessing this:

- Tissue collection (prostate biopsy including original diagnostic and local recurrence sample) to measure the presence of a hypoxia-associated gene signature.
- Urine collection to measure the inflammatory response via damage-associated molecular patterns.
- Blood sample collection (20-30ml) to measures changes in cytokine response following reirradiation and other proteomics analyses.

The long-term storage arrangement for the research data arising from these biological samples will follow The University of Manchester Biobank (site of processing for biological samples) good practice for research guidance on clinical samples. Participants will have the option to consent to making their biological samples available for future research. The biological sample research data will be stored for 20 years once the study has ended.

Baseline and follow-up evaluation

Table 1 shows the full schedule of events.

Table 1: RO-PIP schedule of events.

Time-point / Item	* Baseline	During RT	1 month FU	3 month FU	6 month FU	12 month FU	18 month FU	24 month FU
Informed consent	x							
Registration	x							
Testosterone	x							
Confirmation of eligibility	x							
Randomisation to BT or SBRT	x							
Pre-treatment Prostate Biopsy sample requested and stored	x							
Collection of clinical history and outcome data	x							x
Radiotherapy (BT or SBRT) details	x	x						
PSA test	x			x	x	x	x	x
Functional prostate mpMRI	x		x			x		

PROM assessment (EPIC-26, EORTC QLQ-C30 and IPSS)	x		x	x	x	x		x
Research blood test/ Urine	x		x	x	x			
Clinician reported adverse events		x	x	x	x	x	x	x

Key: FU=follow-up, RT=radiotherapy, BT=brachytherapy, EBRT= External Beam Radiotherapy, mpMRI=multi-parametric magnetic resonance imaging, PROM=patient recorded outcome measure, PSA=prostate specific antigen, EORTC QLQ = European Organization for the Research and Treatment of Cancer Quality of Life Questionnaire, EPIC-26= Expanded Prostate Cancer Index Composite-26, IPSS= international prostate symptom score

Outcomes

Primary outcome

1. Recruitment rates for the whole 24-month recruitment period will be reported overall and per recruiting site. The average recruitment rate per month and in total over the formal monitoring period will be reported.

The study recruitment period is 24 months. To show that patient recruitment targets for a phase III RCT can be met within an adequate timeframe, a "steady state" of recruitment should be observed. In this feasibility study, formal monitoring of recruitment will begin from the start of the patient recruitment where an average of two patients per month must be randomised over the remaining recruitment period in order to demonstrate a "steady state" of recruitment.

Secondary outcomes

1. Incidence of patient reported acute (0-3 months) and long-term toxicity (>3 months) and impact on QoL determined by EPIC-26 (prostate cancer related QoL and functional outcomes), EORTC QLQ-C30 (general QoL score) and international prostate symptom score (IPSS) (urinary and sexual functional outcomes) measurements (Key secondary endpoint).
2. Incidence of clinician-reported treatment toxicity as per Common Terminology Criteria for Adverse Events (CTCAE) version 5.0.
3. Other feasibility endpoints include screening log summaries, treatment and questionnaire compliance, withdrawal rate and reasons for non-recruitment.

Exploratory outcomes

1. MRI biomarkers at 1 month and 1 year post-treatment predictive of toxicity based on PROMs.

For peer review only

2. Hypoxia levels based on a hypoxia associated gene signature obtained from the pre-salvage RT biopsy correlated with MRI biomarkers.
3. Changes in the levels of inflammatory cytokine signatures from urine and blood obtained at baseline and after reirradiation in relation to PROMs.
4. Multiple measures of image quality and reproducibility of prostate functional imaging (e.g. diffusion coefficient values from IVIM sequences) for measuring tumour biology will be summarised.

Sample size

This is an exploratory feasibility study, and there is no informative data in the published literature on which to base a sample size calculation. Therefore, a formal statistical power calculation has not been performed. Feasibility studies are not usually sufficiently powered to provide estimates of effect size, but instead aim to determine the feasibility of specific study aspects and to enable estimation of sample size parameters to inform future studies. For this feasibility study, we plan to recruit 60 patients in total (i.e. 30 to each treatment arm) from three UK hospitals (Leeds Cancer Centre, The Christie and Mount Vernon Cancer Centre), all of which are high-volume tertiary prostate cancer treatment centres. This sample size has been informed by the National Institute of Health Research (NIHR) guidance on feasibility sample sizes and the toxicity outcome data from a recent systematic review[13,24]. Few studies have evaluated PROMs feasibility, establishing the need for this trial[25]. It is estimated that this number of participants will provide an adequate sample to estimate the toxicity rates for the key secondary endpoint in each arm (i.e. 30 per arm)[26,27]. Recruiting 60 patients over 2 years, across 3 centres, would mean an average recruitment rate of 1 patient per centre per month. This information, combined with the estimated toxicity rates from the study, will be used to determine feasibility of a subsequent larger scale RCT.

Recruitment

Sixty patients will be recruited over a 24-month period, approximately 2-3 patients per month (across 3 sites).

Reporting

No formal interim analysis will take place however a study report will be produced for review by the independent data monitoring and ethics committee (DMEC) approximately midway through the study. The aim of the report is to evaluate and monitor the key study objectives (i.e. recruitment rates, number of participants taking up their treatment allocation), as well as expected adverse events (AEs) and serious adverse events (SAEs) and the delivery time of HDR-BT or SBRT post-randomisation.

For peer review only

Data collection, management and analysis

Withdrawal of Participants

Participants who withdraw their consent to the study will be taken off the study. The research team will keep any tissue, blood, urine samples and imaging data already collected and continue analysis (unless the patient requests the destruction of samples and data). Patients will be consented from the outset to continue collecting follow-up data even if patients are withdrawn from the trial, cannot tolerate MRI scans, unable to continue treatment or do not complete all PROMs time points.

Data management

Study data will be managed by the trial coordinator and research fellow under the supervision of the chief investigator and the study statistician.

Data stored on hospital computers will be password protected and in locked rooms in the local hospital radiotherapy and/or radiology (imaging data) departments, only accessible by the local research team. Each patient is assigned a unique patient study ID number at enrolment (based on site and trial number allocated during randomisation step) which will be used on all trial documentation. This pseudoanonymisation step will prevent the patient from being identified by those outside the local research team. The local investigator will keep a subject enrolment and identification log that contains the key to the code, i.e. a record of the personal identification data linked to each patient study ID number.

In compliance with Good Clinical Practice guidelines and in accordance with the University of Leeds Code of Conduct and Research Ethics, the chief or local principal investigator will maintain all records regarding the conduct of the RO-PIP study. These will be securely archived for up to 20 years if required.

Statistical analysis

As this is a feasibility study, it will not involve hypothesis testing to identify whether the intervention has had an impact. Instead, data analysis will be descriptive and involve summary statistics. The analysis of all primary endpoint and all secondary endpoints relating to recruitment and withdrawals from the trial will take place at the end of the 24-month recruitment period. Final analysis of all other endpoint data will be carried out 6 months after the final participant has been randomised.

Future Work

For peer review only

Demonstrating feasibility will facilitate a larger randomised study comparing salvage re-irradiation options with ADT alone, the usual management option. This study would have primary endpoints of survival (overall and metastasis free). This is the first time that a hypoxia gene expression signature will be studied with hypoxia imaging in a prospective cohort of patients with radiorecurrent prostate cancer and could lead to the further studies investigating the introduction in clinical practice of tumour hypoxia testing (from biopsy and/ or imaging) and the biological individualisation of radiotherapy. Given the large number of prostate cancer patients who undergo radiotherapy each year, this would have a significant impact on personalised medicine in the UK.

Trial oversight

A trial management group will be convened for the study, consisting of the chief investigator, principal investigators (for each site), research fellow, trial administrator, and research nurse. The group will meet monthly. The study sponsor (University of Leeds) will monitor the conduct of the trial. A trial report will be produced by the DMEC midway through the study

Ethics and dissemination

This study has been approved by the Yorkshire & The Humber - Bradford Leeds Research Ethics Committee (Reference: 21/YH/0305, IRAS: 297060, January 2022).

The results will be presented in national and international conferences, published in peer-reviewed journals, publicised via social media channels such as twitter, and will be communicated to relevant stakeholders. A plain English report will be shared with the study participants, patients' organisations, PPIE groups and media.

Patient consent for publication

Not applicable.

For peer review only

References

1. Cancer Research UK. Cancer Incidence from Cancer Intelligence Statistical Information Team at Cancer Research UK [Internet]. 2019.
2. Bray F, Ferlay J, Soerjomataram I, Siegel RL, Torre LA, Jemal A. Global cancer statistics 2018: GLOBOCAN estimates of incidence and mortality worldwide for 36 cancers in 185 countries. CA Cancer J Clin [Internet]. 2018;68:394–424.
3. Mottet N, Bellmunt J, Bolla M, Briers E, Cumberbatch MG, De Santis M, et al. EAU-ESTRO-SIOG Guidelines on Prostate Cancer. Part 1: Screening, Diagnosis, and Local Treatment with Curative Intent. Eur Urol [Internet]. 2017;71(4):618–29.
4. Cancer Research UK . Prostate cancer treatment statistics [Internet]. 2017.
5. Beckendorf V, Guerif S, Le Prisé E, Cosset JM, Bougnoux A, Chauvet B, et al. 70 Gy versus 80 Gy in localized prostate cancer: 5-year results of GETUG 06 randomized trial. Int J Radiat Oncol Biol Phys [Internet]. 2011;80(4):1056–63.
6. Zumsteg ZS, Spratt DE, Romesser PB, Pei X, Zhang Z, Kollmeier M, et al. Anatomical patterns of recurrence following biochemical relapse in the dose escalation era of external beam radiotherapy for prostate cancer. J Urol [Internet]. 2015;194(6):1624-1630.
7. Ma TM, Chu F-I, Sandler H, Feng FY, Efstathiou JA, Jones CU, et al. Local Failure Events in Prostate Cancer Treated with Radiotherapy: A Pooled Analysis of 18 Randomized Trials from the Meta-analysis of Randomized Trials in Cancer of the Prostate Consortium (LEVIATHAN). Eur Urol. 2022;
8. Jones CU, Hunt D, McGowan DG, Amin MB, Chetner MP, Bruner DW, et al. Radiotherapy and Short-Term Androgen Deprivation for Localized Prostate Cancer. N Engl J Med. 2011;365(2):107–18.
9. Mbeutcha A, Chauveinc L, Bondiau PY, Chand ME, Durand M, Chevallier D, et al. Salvage prostate re-irradiation using high-dose-rate brachytherapy or focal stereotactic body radiotherapy for local recurrence after definitive radiation therapy. Radiat Oncol [Internet]. 2017;
10. Ingrosso G, Becherini C, Lancia A, Caini S, Ost P, Francolini G, et al. Nonsurgical Salvage Local Therapies for Radiorecurrent Prostate Cancer: A Systematic Review and Meta-analysis. Eur Urol Oncol [Internet]. 2019;3(2):183–97.
11. Chade DC, Eastham J, Graefen M, Hu JC, Karnes RJ, Klotz L, et al. Cancer control and functional outcomes of salvage radical prostatectomy for radiation-recurrent prostate cancer: A systematic review of the literature. Eur Urol [Internet]. 2012;61(5):961–71.
12. Valle LF, Lehrer EJ, Markovic D, Elashoff D, Levin-Epstein R, Karnes RJ, et al. A Systematic Review and Meta-analysis of Local Salvage Therapies After Radiotherapy

For peer review only

for Prostate Cancer (MASTER). Eur Urol. 2020;1(310):1–13.
13. Zhong J, Slevin F, Scarsbrook AF, Serra M, Choudhury A, Hoskin PJ, et al. Salvage Reirradiation Options for Locally Recurrent Prostate Cancer: A Systematic Review. Front Oncol. 2021;11.
14. Cooperberg MR, Vickers AJ, Broering JM, Carroll PR. Comparative risk-adjusted mortality outcomes after primary surgery, radiotherapy, or androgen-deprivation therapy for localized prostate cancer. Cancer [Internet]. 2010;116:5226–5234.
15. Chatzikonstantinou G, Zamboglou N, Rödel C, Zoga E, Strouthos I, Butt SA, et al. High-dose-rate brachytherapy as salvage modality for locally recurrent prostate cancer after definitive radiotherapy: Asystematic review. Strahlentherapie und Onkol [Internet]. 2017;193:683–691.
16. Chitmanee P, Tsang Y, Tharmalingam H, Hamada M, Alonzi R, Ostler P, et al. Single-Dose Focal Salvage High Dose Rate Brachytherapy for Locally Recurrent Prostate Cancer. Clin Oncol [Internet]. 2020;32(4):259-265.
17. Murgic J, Morton G, Loblaw A, D'Alimonte L, Ravi A, Wronski M, et al. Focal Salvage High Dose-Rate Brachytherapy for Locally Recurrent Prostate Cancer After Primary Radiation Therapy Failure: Results From a Prospective Clinical Trial. Int J Radiat Oncol Biol Phys [Internet]. 2018;102(3):561-567.
18. Slevin F, Hodgson S, Rodda SL, Bownes P, Bottomley D, Adiotomre E, et al. Efficacy and toxicity outcomes for patients treated with focal salvage high dose rate brachytherapy for locally recurrent prostate cancer. Clin Transl Radiat Oncol [Internet]. 2020;23:20-26.
19. Hathout L, Mahmoud O, Wang Y, Vergalasova I, Barkati M, Després P, et al. A Phase 2 Randomized Pilot Study Comparing High-Dose-Rate Brachytherapy and Low-Dose-Rate Brachytherapy as Monotherapy in Localized Prostate Cancer. Adv Radiat Oncol [Internet]. 2019;4(4):631-640.
20. Zilli T, Benz E, Dipasquale G, Rouzaud M, Miralbell R. Reirradiation of Prostate Cancer Local Failures After Previous Curative Radiation Therapy: Long-Term Outcome and Tolerance. Int J Radiat Oncol Biol Phys [Internet]. 2016;96:218–322.
21. Pasquier D, Martinage G, Janoray G, Rojas DP, Zerini D, Goupy F, et al. Salvage Stereotactic Body Radiation Therapy for Local Prostate Cancer Recurrence After Radiation Therapy: A Retrospective Multicenter Study of the GETUG. Int J Radiat Oncol Biol Phys [Internet]. 2019;105(4):727-734.
22. Chan A-W, Tetzlaff JM, Altman DG, Laupacis A, Gøtzsche PC, Krleža-Jerić K, et al. SPIRIT 2013 Statement: Defining Standard Protocol Items for Clinical Trials. Ann Intern Med. 2013;158(3):200.
23. Salembier C, Villeirs G, De Bari B, Hoskin P, Pieters BR, Van Vulpen M, et al. ESTRO

For peer review only

ACROP consensus guideline on CT- and MRI-based target volume delineation for primary radiation therapy of localized prostate cancer. Radiother Oncol. 2018;127(1):49–61.

24. Hooper R. Justifying sample size for a feasibility study (NIHR Guidance) [Internet]. 2021.

25. Skolarus TA, Dunn RL, Sanda MG, Chang P, Greenfield TK, Litwin MS, et al. Minimally important difference for the expanded prostate cancer index composite short form. Urology [Internet]. 2015;85(1):101–106.

26. Lancaster GA, Dodd S, Williamson PR. Design and analysis of pilot studies: recommendations for good practice. J Eval Clin Pract. 2004;10(2):307–12.

27. Billingham SA, Whitehead AL, Julious SA. An audit of sample sizes for pilot and feasibility trials being undertaken in the United Kingdom registered in the United Kingdom Clinical Research Network database. BMC Med Res Methodol. 2013;13(1):104.

For peer review only

Figure Legends

Figure 1: Schematic overview of study.

Key – CT = Computed Tomography, PET = positron emission tomography, mpMRI = multi-parametric magnetic resonance imaging, EORTC QLQ = European Organization for the Research and Treatment of Cancer Quality of Life Questionnaire, IPSS = international prostate symptom score, EPIC = Expanded Prostate Cancer Index Composite, QoL = quality of life, CTCAE = Common Terminology Criteria for Adverse Events.

Footnotes

Author Statement/ Contributors:

JZ, SB, MS, PS, PB, CT, MD, DM, CE, RC, YMT, NJT, CW, DB, AS, AC, PH and AH contributed to the study design and protocol development. JZ, AC, PH, AH, SB, PB and CT developed the radiation treatment protocol, and co-wrote the ethics application for the study. JZ, DB, MD, DM, YMT, NJT and AS contributed to the development of the MR imaging aspects. JZ, KR, RR, AC, PH, CW and AH developed the translational biology parts of the study. All co-authors contributed to the writing, critical review and revisions of the manuscript.

Funding:

This work is supported by a CRUK award: grant number 95653118.

Competing interests:

None declared.

Data Availability Statement

Data sharing not applicable as no datasets generated and/or analysed for this study yet.

For peer review only

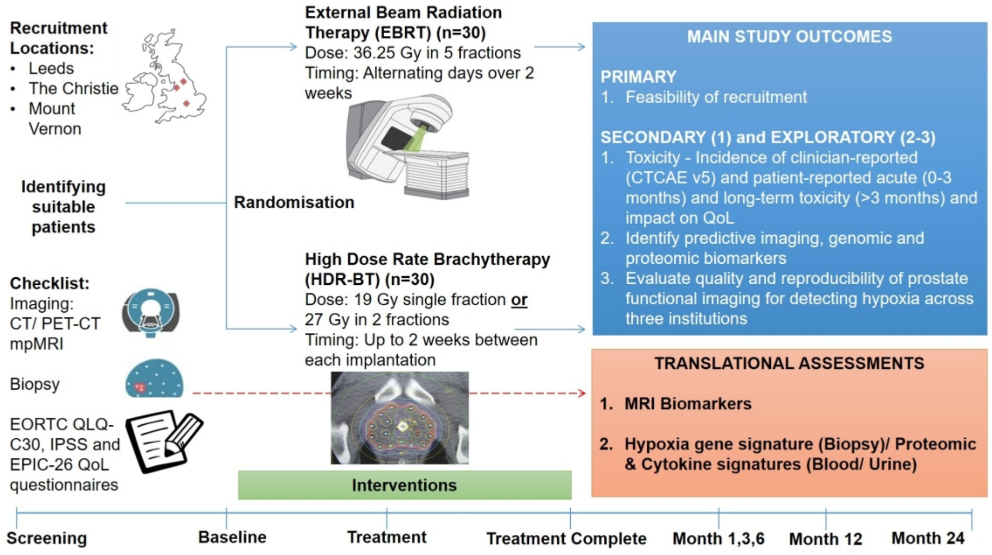

Figure 1: Schematic overview of study.
Key – CT = Computed Tomography, PET = positron emission tomography, mpMRI = multi-parametric magnetic resonance imaging, EORTC QLQ = European Organization for the Research and Treatment of Cancer Quality of Life Questionnaire, IPSS = international prostate symptom score, EPIC = Expanded Prostate Cancer Index Composite, QoL = quality of life, CTCAE = Common Terminology Criteria for Adverse Events.

336x189mm (150 x 150 DPI)

SPIRIT

STANDARD PROTOCOL ITEMS: RECOMMENDATIONS FOR INTERVENTIONAL TRIALS

SPIRIT 2013 Checklist: Recommended items to address in a clinical trial protocol and related documents*

Section/item	ItemNo	Page No	Description
			**Administrative information**
Title	1	1	Descriptive title identifying the study design, population, interventions, and, if applicable, trial acronym
Trial registration	2a	2	Trial identifier and registry name. If not yet registered, name of intended registry
	2b	2	All items from the World Health Organization Trial Registration Data Set
Protocol version	3	1	Date and version identifier
Funding	4		Sources and types of financial, material, and other support
Roles and responsibilities	5a	1	Names, affiliations, and roles of protocol contributors
	5b	1	Name and contact information for the trial sponsor
	5c	1	Role of study sponsor and funders, if any, in study design; collection, management, analysis, and interpretation of data; writing of the report; and the decision to submit the report for publication, including whether they will have ultimate authority over any of these activities
	5d	1	Composition, roles, and responsibilities of the coordinating centre, steering committee, endpoint adjudication committee, data management team, and other individuals or groups overseeing the trial, if applicable (see Item 21a for data monitoring committee)
**Introduction**			
Background and rationale	6a	4	Description of research question and justification for undertaking the trial, including summary of relevant studies (published and unpublished) examining benefits and harms for each intervention

For peer review only

	6b	4	Explanation for choice of comparators
Objectives	7	2	Specific objectives or hypotheses
Trial design	8	2	Description of trial design including type of trial (eg, parallel group, crossover, factorial, single group), allocation ratio, and framework (eg, superiority, equivalence, noninferiority, exploratory)
			**Methods: Participants, interventions, and outcomes**
Study setting	9	6	Description of study settings (eg, community clinic, academic hospital) and list of countries where data will be collected. Reference to where list of study sites can be obtained
Eligibility criteria	10	6	Inclusion and exclusion criteria for participants. If applicable, eligibility criteria for study centres and individuals who will perform the interventions (eg, surgeons, psychotherapists)
Interventions	11a	7	Interventions for each group with sufficient detail to allow replication, including how and when they will be administered
	11b	6	Criteria for discontinuing or modifying allocated interventions for a given trial participant (eg, drug dose change in response to harms, participant request, or improving/worsening disease)
	11c	7	Strategies to improve adherence to intervention protocols, and any procedures for monitoring adherence (eg, drug tablet return, laboratory tests)
	11d	7	Relevant concomitant care and interventions that are permitted or prohibited during the trial
Outcomes	12	10/11	Primary, secondary, and other outcomes, including the specific measurement variable (eg, systolic blood pressure), analysis metric (eg, change from baseline, final value, time to event), method of aggregation (eg, median, proportion), and time point for each outcome. Explanation of the clinical relevance of chosen efficacy and harm outcomes is strongly recommended
Participant timeline	13	9/10	Time schedule of enrolment, interventions (including any run-ins and washouts), assessments, and visits for participants. A schematic diagram is highly recommended (see Figure)

For peer review only - http://bmjopen.bmj.com/site/about/guidelines.xhtml

Sample size	14	12	Estimated number of participants needed to achieve study objectives and how it was determined, including clinical and statistical assumptions supporting any sample size calculations
Recruitment	15	12	Strategies for achieving adequate participant enrolment to reach target sample size
			**Methods: Assignment of interventions (for controlled trials)**
Allocation:			
Sequence generation	16a	12	Method of generating the allocation sequence (eg, computer-generated random numbers), and list of any factors for stratification. To reduce predictability of a random sequence, details of any planned restriction (eg, blocking) should be provided in a separate document that is unavailable to those who enrol participants or assign interventions
Allocation concealment mechanism	16b	12	Mechanism of implementing the allocation sequence (eg, central telephone; sequentially numbered, opaque, sealed envelopes), describing any steps to conceal the sequence until interventions are assigned
Implementation	16c	12	Who will generate the allocation sequence, who will enrol participants, and who will assign participants to interventions
Blinding (masking)	17a	n/a	Who will be blinded after assignment to interventions (eg, trial participants, care providers, outcome assessors, data analysts), and how
	17b	n/a	If blinded, circumstances under which unblinding is permissible, and procedure for revealing a participant's allocated intervention during the trial
			**Methods: Data collection, management, and analysis**

Data collection methods	18a	12/13	Plans for assessment and collection of outcome, baseline, and other trial data, including any related processes to promote data quality (eg, duplicate measurements, training of assessors) and a description of study instruments (eg, questionnaires, laboratory tests) along with their reliability and validity, if known. Reference to where data collection forms can be found, if not in the protocol
	18b	13	Plans to promote participant retention and complete follow-up, including list of any outcome data to be collected for participants who discontinue or deviate from intervention protocols
Data management	19	13	Plans for data entry, coding, security, and storage, including any related processes to promote data quality (eg, double data entry; range checks for data values). Reference to where details of data management procedures can be found, if not in the protocol
Statistical methods	20a	13	Statistical methods for analysing primary and secondary outcomes. Reference to where other details of the statistical analysis plan can be found, if not in the protocol
	20b	14	Methods for any additional analyses (eg, subgroup and adjusted analyses)
	20c	13/14	Definition of analysis population relating to protocol non-adherence (eg, as randomised analysis), and any statistical methods to handle missing data (eg, multiple imputation)
			**Methods: Monitoring**
Data monitoring	21a	13	Composition of data monitoring committee (DMC); summary of its role and reporting structure; statement of whether it is independent from the sponsor and competing interests; and reference to where further details about its charter can be found, if not in the protocol. Alternatively, an explanation of why a DMC is not needed
	21b	12	Description of any interim analyses and stopping guidelines, including who will have access to these interim results and make the final decision to terminate the trial

For peer review only - http://bmjopen.bmj.com/site/about/guidelines.xhtml

Harms	22	12	Plans for collecting, assessing, reporting, and managing solicited and spontaneously reported adverse events and other unintended effects of trial interventions or trial conduct
Auditing	23	12	Frequency and procedures for auditing trial conduct, if any, and whether the process will be independent from investigators and the sponsor
			**Ethics and dissemination**
Research ethics approval	24	2	Plans for seeking research ethics committee/institutional review board (REC/IRB) approval
Protocol amendments	25	12	Plans for communicating important protocol modifications (eg, changes to eligibility criteria, outcomes, analyses) to relevant parties (eg, investigators, REC/IRBs, trial participants, trial registries, journals, regulators)
Consent or assent	26a	6	Who will obtain informed consent or assent from potential trial participants or authorised surrogates, and how (see Item 32)
	26b	6	Additional consent provisions for collection and use of participant data and biological specimens in ancillary studies, if applicable
Confidentiality	27	13	How personal information about potential and enrolled participants will be collected, shared, and maintained in order to protect confidentiality before, during, and after the trial
Declaration of interests	28	18	Financial and other competing interests for principal investigators for the overall trial and each study site
Access to data	29	14	Statement of who will have access to the final trial dataset, and disclosure of contractual agreements that limit such access for investigators
Ancillary and post-trial care	30	19	Provisions, if any, for ancillary and post-trial care, and for compensation to those who suffer harm from trial participation

For peer review only

Dissemination policy	31a	15	Plans for investigators and sponsor to communicate trial results to participants, healthcare professionals, the public, and other relevant groups (eg, via publication, reporting in results databases, or other data sharing arrangements), including any publication restrictions
	31b	15	Authorship eligibility guidelines and any intended use of professional writers
	31c	15	Plans, if any, for granting public access to the full protocol, participant-level dataset, and statistical code
**Appendices**			
Informed consent materials	32	Available	Model consent form and other related documentation given to participants and authorised surrogates
Biological specimens	33	14	Plans for collection, laboratory evaluation, and storage of biological specimens for genetic or molecular analysis in the current trial and for future use in ancillary studies, if applicable

*It is strongly recommended that this checklist be read in conjunction with the SPIRIT 2013 Explanation & Elaboration for important clarification on the items. Amendments to the protocol should be tracked and dated. The SPIRIT checklist is copyrighted by the SPIRIT Group under the Creative Commons "Attribution-NonCommercial-NoDerivs 3.0 Unported" license.

For peer review only
