# Supplementary material for: Reirradiation Options for Previously Irradiated Prostate cancer (RO-PIP): Feasibility study investigating toxicity outcomes following reirradiation with stereotactic body radiotherapy (SBRT) versus high-dose-rate brachytherapy (HDR-BT)
Source: BMJ Open. 2022 Nov 8;12(11):e068580. doi: 10.1136/bmjopen-2022-068580 (PMC9644357; doi:10.1136/bmjopen-2022-068580)
Supplement: Reviewer comments [file bmjopen-2022-068580.reviewer_comments.pdf]

PEER REVIEW HISTORY

BMJ Open publishes all reviews undertaken for accepted manuscripts. Reviewers are asked to complete a checklist review form (**http://bmjopen.bmj.com/site/about/resources/checklist.pdf**) and are provided with free text boxes to elaborate on their assessment. These free text comments are reproduced below.

ARTICLE DETAILS

**TITLE (PROVISIONAL)**	Reirradiation Options for Previously Irradiated Prostate cancer (RO-PIP): Feasibility study investigating toxicity outcomes following reirradiation with stereotactic body radiotherapy (SBRT) vs. high dose-rate brachytherapy (HDR-BT)
**AUTHORS**	Zhong, Jim; Brown, Sarah; Serra, Maria; Shuttleworth, Pam; Bownes, Peter; Thompson, Christopher; Reed, Rachel; Reeves, Kimberley; Dubec, Michael; McHugh, Damien; Eccles, Cynthia; Chuter, Robert; Tsang, Yat Man; Taylor, N. Jane; West, Catharine; Buckley, David; Scarsbrook, Andrew; Choudhury, Ananya; Hoskin, Peter; Henry, Ann

This article was not externally reviewed at BMJ Open. Protocols that have been independently assessed prior to submission to BMJ Open are usually fast-tracked to publication on the grounds that further substantial changes will not be possible. This independent assessment will usually be external, independent review for both a substantial grant award from a non-commercial or government funder and ethics approval.
